# Inference of Gene Flow between Species under Misspecified Models

**DOI:** 10.1093/molbev/msac237

**Published:** 2022-11-01

**Authors:** Jun Huang, Yuttapong Thawornwattana, Tomáš Flouri, James Mallet, Ziheng Yang

**Affiliations:** School of Biomedical Engineering, Capital Medical University, Beijing 100069, P.R. China; Department of Organismic and Evolutionary Biology, Harvard University, Cambridge, MA 02138; Department of Genetics, Evolution and Environment, University College London, London WC1E 6BT, United Kingdom; Department of Organismic and Evolutionary Biology, Harvard University, Cambridge, MA 02138; Department of Genetics, Evolution and Environment, University College London, London WC1E 6BT, United Kingdom

**Keywords:** gene flow, model misspecification, multispecies coalescent, introgression, Bayesian phylogenetics and phylogeography (BPP), species tree

## Abstract

Genomic sequence data provide a rich source of information about the history of species divergence and interspecific hybridization or introgression. Despite recent advances in genomics and statistical methods, it remains challenging to infer gene flow, and as a result, one may have to estimate introgression rates and times under misspecified models. Here we use mathematical analysis and computer simulation to examine estimation bias and issues of interpretation when the model of gene flow is misspecified in analysis of genomic datasets, for example, if introgression is assigned to the wrong lineages. In the case of two species, we establish a correspondence between the migration rate in the continuous migration model and the introgression probability in the introgression model. When gene flow occurs continuously through time but in the analysis is assumed to occur at a fixed time point, common evolutionary parameters such as species divergence times are surprisingly well estimated. However, the time of introgression tends to be estimated towards the recent end of the period of continuous gene flow. When introgression events are assigned incorrectly to the parental or daughter lineages, introgression times tend to collapse onto species divergence times, with introgression probabilities underestimated. Overall, our analyses suggest that the simple introgression model is useful for extracting information concerning between-specific gene flow and divergence even when the model may be misspecified. However, for reliable inference of gene flow it is important to include multiple samples per species, in particular, from hybridizing species.

## Introduction

Hybridization can enhance variation in recipient species, and has long been recognized as an important process in plants that can stimulate the origin of new species (e.g., Anderson 1949; Mallet 2007). Analyses of genomic data in the past decade have highlighted the prevalence of hybridization or introgression in animals as well, including bears (Liu et al. 2014; Kumar et al. 2017), birds (Ellegren et al. 2012), and butterflies (Martin et al. 2013). Between-species gene flow may involve either sister or non-sister species and may play an important role in ecological adaptation (Mallet et al. 2016; Martin and Jiggins 2017). Gene flow can be a major contributor of genealogical variation across the genome and gene tree-species tree discordance, in addition to ancestral polymorphism or delayed coalescence (Maddison 1997; Nichols 2001).

There is a long history of studies in population genetics of models of population subdivision and migration (Wright 1943; Malecot 1948; Slatkin 1987), and a number of methods have been developed to estimate the migration rate between populations (Beerli and Felsenstein 1999, 2001; Bahlo and Griffiths 2000). An important limitation of models of population subdivision, when applied to data from different species or subspecies, is that they do not account for the divergence history of the populations or species. Introducing a population/species phylogeny into models of population subdivision not only improves the realism of the model but also opens up opportunities for addressing a number of interesting questions in evolutionary biology, such as estimating species divergence times and ancestral population sizes, delineating species boundaries, and inferring the direction, rate, and timing of gene flow (Jiao et al. 2021).

Two classes of models of gene flow have been developed that accommodate the phylogeny of the species, both of which are extensions of the multispecies coalescent (MSC) model (Rannala and Yang 2003). The first is the MSC-with-migration model (MSC-M, or isolation-with-migration or IM model, Hey and Nielsen 2004; Hey 2010; Zhu and Yang 2012; Dalquen et al. 2017; Hey et al. 2018), which assumes that two species exchange migrants at a certain rate over an extended time period. The rate of gene flow from species *A* to *B* is measured by the proportion of migrants ($m_{AB}$) in the receiving population *B* or by the population migration rate, $M_{AB}=N_{B}m_{AB}$, the expected number of immigrants from *A* to *B* per generation, where $N_{B}$ is the (effective) population size of species *B*. We note that the isolation-with-initial-migration (IIM) model of Costa and Wilkinson-Herbots (2017), which assumes that gene flow occurs initially after species divergence but stops after a period of time when reproductive isolation has been fully established, is an instance of the MSC-M or IM model (see below). The second class of models of gene flow is the MSC-with-introgression (MSci) model (Flouri et al. 2020), also known as multispecies network coalescent model (MSNC; Wen and Nakhleh 2018; Zhang et al. 2018), which assumes that gene flow occurs at fixed time points in the past. The rate of gene flow is measured by the introgression probability (φ or γ), which is the proportion of successful immigrants in the population at the time of introgression.

In the real world, introgressed alleles may be removed by natural selection because they are involved in hybrid incompatibility and are deleterious in the genetic background of the recipient population (Dobzhansky 1937; Muller 1942) or because they are linked to such loci (Petry 1983; Barton and Bengtsson 1986; Uecker et al. 2015). Thus the rate of gene flow (*M* in MSC-M or φ in MSci), when those models are used to analyze genomic sequence data, reflect the long-term effects of selection and drift as well as hybridization or introgression (Martin and Jiggins 2017). Such an effective rate of gene flow may be expected to vary across the genome, influenced by the presence of loci in the genomic region important in ecological adaptation and by the local recombination rate (Bürger and Akerman 2011; Aeschbacher and Bürger 2014; Akerman and Bürger 2014; Schumer et al. 2018; Edelman et al. 2019; Martin et al. 2019). The rate may also vary over time, depending on geological or ecological events that cause changes in the ecology and distribution of the species and in the chance for two species to exchange genes. One can envisage models of gene flow in which the rate varies over time and across genomic regions. For the present, such extended models are not yet implemented in the MSC framework, and the feasibility of fitting such parameter-rich models to genomic datasets is unexplored. MSC-M and MSci models implemented to date (Dalquen et al. 2017; Hey et al. 2018; Wen and Nakhleh 2018; Zhang et al. 2018; Flouri et al. 2020) assume constant rates, and should be considered first approximations when applied to genomic sequence data.

In this paper, we use mathematical analysis and computer simulation to examine the impact of model misspecification on estimation of parameters under the MSci model, such as species divergence and introgression times, population sizes, and introgression probabilities. We use the Bayesian program Bayesian phylogenetics and phylogeography (BPP) (Flouri et al. 2018, 2020) to analyze multilocus sequence data simulated under various MSci and MSC-M models. Although Bpp is our own implementation of the MSci model, our results should apply to similar exact or likelihood methods (Wen and Nakhleh 2018; Zhang et al. 2018). Our results may also apply to approximate methods, which use summaries of the data such as the genome-wide site-pattern counts (as in the *D*-statistic, Green et al. 2010 and Hyde, Meng and Kubatko 2009; Blischak et al. 2018), reconstructed gene trees (as in Snaq, Solis-Lemus and Ane 2016), or other summary statistics used in Approximate Bayesian Computation (Dittberner et al. 2022). However, approximate methods do not make a full use of information in the data and may not identify all parameters in the model. For example, the *D*-statistic is agnostic of the mode of gene flow (migration versus introgression) and cannot be applied to data sampled from only two species or populations. The computational strengths and statistical weaknesses of approximate methods have been discussed by a number of authors (Degnan 2018; Elworth et al. 2019; Jiao et al. 2021; Zhu and Yang 2021; Hibbins and Hahn 2022; Ji et al. 2022). In contrast, likelihood methods integrate over all possible gene trees underlying the sequence alignments, making use of all information about the model and parameters in the sequence data. They typically involve a heavy computational load. However, recent algorithmic improvements have made it possible to apply the MSci model to genome-scale datasets with more than 10,000 loci (Flouri et al. 2020). Inferring introgression events or constructing an introgression model using genomic sequence data, however, remains a challenging task, even when a binary species tree is specified, onto which introgression events can be added (Ji et al. 2022; Thawornwattana et al. 2022); see Discussion for an overview of currently available methods for inferring gene flow on a species phylogeny. For these and many other reasons, the model of gene flow assumed in our data analysis may often be incorrect. An important question is to what extent inference of gene flow and estimation of the timing and rate of gene flow can still be achieved when the model of gene flow is misspecified. The impact of model misspecification on estimation of other evolutionary parameters such as species divergence times is also of major concern.

Although there are many ways in which the assumed model is wrong, we are particularly interested in a few types that are likely in real data analyses (Finger et al. 2022; Thawornwattana et al. 2022). First, gene flow may be occurring continuously during a time period but an MSci model is fitted to the genomic data, which assumes that gene flow occurred at a particular time point (e.g., Wen and Nakhleh 2018; Jiao et al. 2020). We are here interested in whether species divergence times and ancestral population sizes are affected by the misspecification, and how the migration rate in the migration model (*M*) corresponds to the introgression probability in the MSci model (φ). The case of two species is analytically tractable. We study the limit of the maximum-likelihood estimates (MLEs) of introgression probability and introgression time when the data size (the number of loci) approaches infinity when the data are generated under the MSC-M model. We use computer simulation to verify and extend the analytical calculation.

Second, the introgression event may be assigned to a wrong branch on the species tree, for example, to a parental or daughter branch of the genuine introgression lineage. Alternatively, introgression may involve species that have since gone extinct or are not included in the data sample. The presence of such ghost species is known to mislead inference of the history of gene flow for the sampled species (Beerli 2004; Tricou et al. 2022). Thus we conducted simulation to examine the impact of unsampled species on the inference of gene flow. In general, our results demonstrate the usefulness of the simple introgression model in inferring gene flow using genomic sequence data.

## Results

### Correspondence between the MSC-M and MSci Models in the Case of two Species

#### Notation and Definition of Parameters

**Fig. 1.**
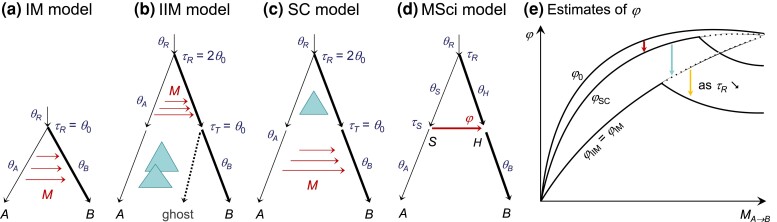
(*a*–*c*) Three MSC-M models for two species *A* and *B* used to generate data: IM (isolation with migration), IIM (isolation with initial migration), and SC (secondary contact). The IIM model is an instance of the MSC-M model with a ghost species at node *T* and with migration from species *A* to *T* (*b*). Similarly, the SC model (*c*) is a case of the MSC-M model with $\tau_{T}>0$. Note that $\tau_{T}$ is the time when migration stopped in the IIM model and the time when migration started in the SC model. In the numerical calculations and in the simulations, we assumed the population size $\theta_{0}=0.002$ for the thin branches and $\theta_{1}=0.01$ for the thick branches, and the migration rate was $M_{AB}=0.2$ migrants from *A* to *B* per generation. Note that in our setup, the time period of gene flow is $\Delta \tau =\theta_{0}$ in all three models. (*d*) The introgression (MSci) model used to analyze the data. (*e*) A schematic summary of the estimate of the introgression probability (φ) in the MSci model (*d*) when the data are generated under the MSC-M models of *a*–*c*. The sudden drop in $\hat{\varphi }$ as $M_{AB}$ increases coincides with an underestimation of $\tau_{R}$ and overestimation of $\theta_{R}$.

Following Jiao et al. (2020), we study the asymptotic behavior of Bayesian parameter estimation under the introgression (MSci) model when the data are generated under the migration (IM) model in the case of two species, with one sequence per species per locus (fig. 1). Here we focus on this simple case because it is analytically tractable. Note that our Bayesian implementation in Bpp (Flouri et al. 2020) accommodates an arbitrary number of species and an arbitrary number of sequences per species per locus, and the likelihood calculation averages over the gene genealogy for the sequences at each locus. We assume an infinite number of loci, and the data at each locus consist of a pair of sequences (a,b) from the two species, with *x* differences at *n* sites. The coalescent time *t* for the locus is unknown and underlies the observed difference. Jiao et al. (2020) analyzed the MSC-M model (fig. 1*a*) assuming infinite sequence length (n=∞) so that the true coalescent time between the two sequences (*t*) is known. Here we accommodate random fluctuations in the number of mutations due to finite sequence length and consider three variants of the migration model.

In the basic IM model (fig. 1*a*), species *A* and *B* diverged at time $\tau_{R}$ and there has since been gene flow from *A* to *B* at the rate of $M_{AB}$ migrants per generation. The IIM model (fig. 1*b*) assumes that migration occurred initially after species divergence but stopped at time $\tau_{T}>0$ (Costa and Wilkinson-Herbots 2017), and is represented by an MSC-M model for three species including a ghost species. Here the ghost does not necessarily represent a real species but is a mathematical device for specifying the IIM model. The IIM model becomes identical to the IM model when $\tau_{T}=0$. We also consider a secondary contact (SC) model (fig. 1*c*), in which two species initially had complete isolation but came into contact at a certain time point ($\tau_{T}$) with ongoing gene flow at the rate of $M_{AB}$ ever since (Costa and Wilkinson-Herbots 2021). This is similarly specified using a ghost species at time point $\tau_{T}$ (fig. 1*c*). The migration model involves three types of parameters: species divergence times ($\tau_{R},\tau_{T}$), population sizes for extant, and extinct species ($\theta_{A},\theta_{B},\theta_{T},\theta_{R}$), and the (population) migration rate $M_{AB}$. The population size parameter for any species with (effective) population size *N* is defined as θ=4Nμ, where μ is the mutation rate per site per generation. We refer to a branch on the species tree by its daughter node so that branch *RA* is also branch *A*, with population size parameter $\theta_{A}$. Both divergence times (τ) and population sizes (θ) are measured by the expected number of mutations per site.

#### Asymptotic Theory

We first consider the IIM model of figure 1*b*, of which the IM model of figure 1*a* is a special case with $\tau_{T}=0$. The backwards-in-time process of coalescent and migration in time interval $\left(\tau_{T},\tau_{R}\right)$ is described by a Markov chain with three states: *AB*, *AA*, and *A* (Notohara 1990). Here *AB* is the initial state, with two sequences in the sample, one in *A* and another in *B*; *AA* means both sequences are in *A* (in other words, sequence *b* is traced back into *A*); and *A* means one sequence in *A* (in other words, sequence *b* is traced back into *A* and has coalesced with sequence *a*). Note that in the Markov chain, time runs backwards, so the transition from *AB* to *AA* means migration of a sequence from *A* to *B* in the real world. The generator matrix for the Markov chain is (see, e.g., Notohara 1990; Jiao et al. 2020)

(1) $$Q=\begin{array}{cccc} & AB & AA & A\\ AB & -w & w & 0\\ AA & 0 & -\frac{2}{\theta_{A}} & \frac{2}{\theta_{A}}\\ A & 0 & 0 & 0 \end{array}$$

where $w=m_{AB}/\mu =4M_{AB}/\theta_{B}$ is the *mutation-scaled migration rate*, and $2/\theta_{A}$ is the coalescent rate in population *A*, with one time unit being the expected time taken to accumulate one mutation per site. *Q* has eigenvalues $\lambda_{1}=0$, $\lambda_{2}=-2/\theta_{A}$, and $\lambda_{3}=-w$.

Let the transition probability matrix over time *t* be $P(t)=\{p_{ij}(t)\}=e^{Qt}$, where $p_{ij}(t)$ is the probability that the Markov chain will be in state *j* time *t* later given that it is in state *i* at time 0. This is

(2) $$P(t)=\left[\begin{array}{ccc} e^{-wt} & \frac{\theta_{A}w}{2-\theta_{A}w}(e^{-wt}-e^{-\frac{2}{\theta_{A}}t}) & 1-\frac{2e^{-wt}-\theta_{A}we^{-\frac{2}{\theta_{A}}t}}{2-\theta_{A}w}\\ 0 & e^{-\frac{2}{\theta_{A}}t} & 1-e^{-\frac{2}{\theta_{A}}t}\\ 0 & 0 & 1 \end{array}\right].$$

The probability density of coalescent time *t* is thus

(3) $$\begin{array}{ll} & f_{\mathrm{iim}}(t)=\left\{ \begin{array}{ll} P_{AB,AA}(t-\tau_{T})\frac{2}{\theta_{A}}, & \mathrm{if}\;\tau_{T}<t<\tau_{R},\\ {}[1-P_{AB,A}(\tau_{R}-\tau_{T})]\frac{2}{\theta_{R}}\;e^{-\frac{2}{\theta_{R}}(t-\tau_{R})}, & \mathrm{if}\;t>\tau_{R} \end{array}\right.\\ & \;=\left\{ \begin{array}{l} \frac{2w}{2-\theta_{A}w}[e^{-w(t-\tau_{T})}-e^{-\frac{2}{\theta_{A}}(t-\tau_{T})}],\\ \;\mathrm{if}\;\tau_{T}<t<\tau_{R},\\ \left[\frac{2}{2-\theta_{A}w}e^{-w(\tau_{R}-\tau_{T})}-\frac{\theta_{A}w}{2-\theta_{A}w}e^{-\frac{2}{\theta_{A}}(\tau_{R}-\tau_{T})}\right]\frac{2}{\theta_{R}}e^{-\frac{2}{\theta_{R}}(t-\tau_{R})},\\ \;\mathrm{if}\;t>\tau_{R}. \end{array}\right. \end{array}$$

This is a function of $w=4M_{AB}/\theta_{B}$ but not of $M_{AB}$ and $\theta_{B}$ individually. The parameters specifying the density are thus $\Theta_{\mathrm{iim}}=(w,\theta_{A},\theta_{R},\tau_{R},\tau_{T})$. Note that the density under the IM model $f_{\mathrm{im}}(t)$ is given by $f_{\mathrm{iim}}(t)$ with $\tau_{T}=0$ (fig. 1*b*and*c*).

Similarly under the secondary-contact (SC) model (fig. 1*c*), the coalescent-with-migration process over the time interval (0, $\tau_{T}$) is described by the Markov chain of equation (1). Given the parameters $\Theta_{m}$, the probability density of coalescent time *t* is

(4) $$\begin{array}{ll} \;f_{\mathrm{sc}}(t) & =\left\{ \begin{array}{ll} P_{AB,AA}(t)\frac{2}{\theta_{A}}, & \mathrm{if}\;0<t<\tau_{T},\\ P_{AB,AA}(\tau_{T})\frac{2}{\theta_{A}}\;e^{-\frac{2}{\theta_{A}}(t-\tau_{T})}, & \mathrm{if}\;\tau_{T}<t<\tau_{R},\\ \left[P_{AB,AA}(\tau_{T})\frac{2}{\theta_{A}}\;e^{-\frac{2}{\theta_{A}}(\tau_{R}-\tau_{T})}+P_{AB,AB}(\tau_{T})\right]\\ \;\times \frac{2}{\theta_{R}}\;e^{-\frac{2}{\theta_{R}}(t-\tau_{R})}, & \mathrm{if}\;t>\tau_{R} \end{array}\right.\\ & =\left\{ \begin{array}{ll} \frac{w\theta_{A}}{2-w\theta_{A}}[e^{-wt}-e^{-\frac{2}{\theta_{A}}t}]\frac{2}{\theta_{A}}, & \mathrm{if}\;0<t<\tau_{T},\\ \frac{w\theta_{A}}{2-w\theta_{A}}[e^{-w\tau_{T}}-e^{-\frac{2}{\theta_{A}}\tau_{T}}]\frac{2}{\theta_{A}}e^{-\frac{2}{\theta_{A}}(t-\tau_{T})}, & \mathrm{if}\;\tau_{T}<t<\tau_{R},\\ \left[\frac{w\theta_{A}}{2-w\theta_{A}}[e^{-w\tau_{T}}-e^{-\frac{2}{\theta_{A}}\tau_{T}}]e^{-\frac{2}{\theta_{A}}(\tau_{R}-\tau_{T})}+e^{-w\tau_{T}}\right]\\ \;\times \frac{2}{\theta_{R}}e^{-\frac{2}{\theta_{R}}(t-\tau_{R})}, & \mathrm{if}\;t>\tau_{R}. \end{array}\right. \end{array}$$

Under the MSci model (fig. 1*d*), we have (e.g., Jiao et al. 2020)

(5) $$f_{i}(t)=\left\{ \begin{array}{ll} \varphi \frac{2}{\theta_{S}}e^{-\frac{2}{\theta_{S}}(t-\tau_{S})}, & \mathrm{if}\;\tau_{S}<t<\tau_{R},\\ {}[\varphi e^{-\frac{2}{\theta_{S}}(\tau_{R}-\tau_{S})}+(1-\varphi )]\frac{2}{\theta_{R}}e^{-\frac{2}{\theta_{R}}(t-\tau_{R})}, & \mathrm{if}\;t>\tau_{R}. \end{array}\right.$$

This is a function of parameters $\Theta_{i}=(\varphi ,\theta_{R},\theta_{S},\tau_{R},\tau_{S})$. Given the coalescent time *t* for a locus, the probability of observing *x* differences at *n* sites under the JC mutation model (Jukes and Cantor 1969) is given by the binomial probability

(6) $$f(x\;|\;t)=(\frac{3}{4}-\frac{3}{4}e^{-\frac{8}{3}t}{)}^{x}\cdot (\frac{1}{4}+\frac{3}{4}e^{-\frac{8}{3}t}{)}^{n-x}.$$

The marginal probability of observing *x* differences at *n* sites, under both the migration (IM, IIM, SC) and introgression (MSci) models, is

(7) $$f(x\;|\;\Theta )=\int_{0}^{\infty }f(x\;|\;t)f(t\;|\;\Theta )\;dt,$$

where f(t|Θ) is given by equations (3), (4), or (5).

For analytical tractability of the likelihood (eq. 7), we assume the infinite-sites mutation model instead of JC, and replace the binomial likelihood by a Poisson approximation

(8) $$f(x\;|\;t)=\frac{1}{x!}(2nt{)}^{x}\;e^{-2nt}.$$

Equation (7) is derived in SI text, as supplementary equation (S6), Supplementary material online for the IM (with $\tau_{T}=0$) and IIM (with $\tau_{T}>0$) models, supplementary equation (S7), Supplementary material online for the SC model, and supplementary equation (S9), Supplementary material online for the MSci model.

Suppose the data are generated under the migration model (IM, IIM, or SC) and analyzed under the MSci model. When the number of loci L→∞, the MLE ${\hat{\Theta }}_{i}$ under MSci will converge to $\Theta_{i}^{*}$, which minimizes the Kullback–Leibler (KL) divergence

(9) $$D(\Theta_{m}∥\Theta_{i})=\sum_{x=0}^{n}f_{m}(x\;|\;\Theta_{m})\log\;\frac{\;f_{m}(x\;|\;\Theta_{m})}{f_{i}(x\;|\;\Theta_{i})},$$

where the subscript “m” stands for any of the three MSC-M models (“im” for IM, “iim” for IIM, or “sc” for SC, fig. 1*a*–*c*). The KL divergence is a measure of distance from the fitting introgression model to the true migration model: here $\Theta_{m}$ are fixed, whereas $\Theta_{i}$ are being estimated. The limiting values $\Theta_{i}^{*}$ as L→∞ are also known as the *pseudo-true parameter values* for the misspecified MSci model. The BFGS optimization routine in Paml (Yang 2007) is used to minimize equation (9) to obtain the MLEs.

We are in particular interested in the introgression probability φ and the introgression time $\tau_{S}$. Note that under the migration model, the probability that any lineage from species *B* traces back to *A* is

(10) $$\varphi_{0}=1-e^{-4M_{AB}\Delta \tau /\theta_{B}},$$

where Δτ is the time period of gene flow (fig. 1*a*–*c*). Equation (10) gives the expected proportion of migrants under the true migration model. When $M_{AB}$ is small, $\varphi_{0}\approx (4M_{AB}/\theta_{B})\Delta \tau$, which is also given by equating the expected total number of migrants under the two models: $N_{B}\varphi_{0}\approx m_{AB}N_{B}\Delta \tau /\mu$. Note that $m_{AB}N_{B}$ is the expected number of migrants per generation and Δτ/μ is the number of generations with gene flow.

It may be noted that the theory of equation (9) can be used to study the limiting parameter estimates (when L→∞) in the migration model when the true model is the introgression model. One has only to flip the roles of $f_{m}(x\;|\;\Theta_{m})$ and $f_{i}(x\;|\;\Theta_{i})$ in equation (9). This is not pursued in this paper.

#### Asymptotic Results under the IM Model

**Fig. 2.**
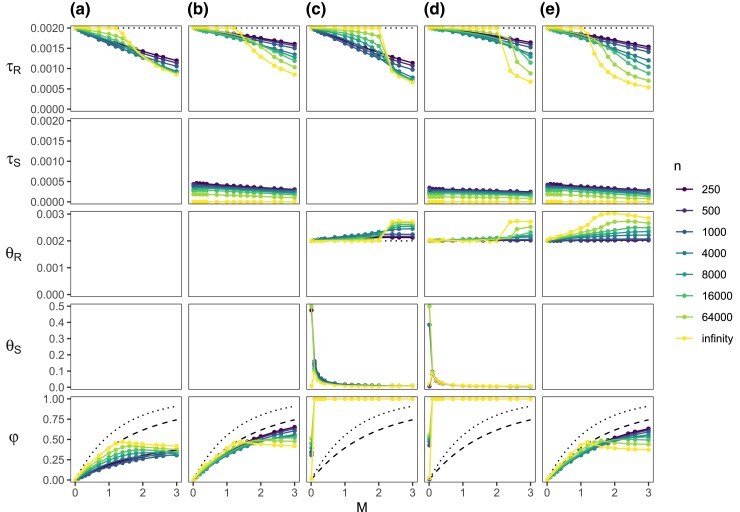
Best-fitting parameter values under the MSci model of figure 1*d* when data of two sequences per locus (one per species), each of *n* sites, are generated under the IM model of figure 1*a*. Five methods (a-e) are used to fit the MSci model, estimating 2, 3, 4, 5, and 4 parameters, respectively, whereas the other parameters are fixed. In (*a*), $\tau_{R}$ and φ are estimated, but $\theta_{R}$ and $\theta_{S}$ are fixed at their true values in the IM model, and the introgression time $\tau_{S}=\tau_{H}$ is fixed at $\tau_{T}=0$. In (*b*), $\tau_{S}$ is estimated as well. In (*c*), $\tau_{S}=0$ is fixed, whereas the other four parameters are estimated. In (*d*), all five parameters are estimated. In (*e*), the constraint $\theta_{R}=\theta_{S}$ is enforced so that four free parameters are estimated. The dotted lines for φ indicate the true total amount of introgression of equation (10). The dashed lines indicate $\varphi^{*}$ of equation (11). The true and best-fitting distributions of the coalescent time (*t*) are shown in supplementary figure S1, Supplementary Material online.

We used the asymptotic theory (eq. 9) to obtain the MLEs ($\Theta_{i}^{*}$) under the MSci model (fig. 1*d*) when the data consist of an infinite number of loci, with one sequence of length *n* per species per locus, generated under the IM, IIM, or SC models (fig. 1*a*–*c*). The true parameter values used ($\Theta_{m}$) are shown in figure 1. The MLEs $\Theta_{i}^{*}$ are shown in figure 2 and the true and best-fitting distributions of the coalescent time *t* are shown in supplementary figure S1, Supplementary Material online for the IM model. The corresponding results for the IIM and SC models are in supplementary figures S2–S5, Supplementary Material online, to be discussed in the next sections.

We use five methods (a–e) to fit the MSci model, with method d estimating all five parameters, whereas the others have some parameters fixed (fig. 2). We examined the effects of the sequence length (*n*) and the migration rate ($M_{AB}$). Note that five parameters are identifiable under the MSci model: $\Theta_{i}=(\tau_{R},\tau_{S},\theta_{R},\theta_{S},\varphi )$ (fig. 1*d*), and $\theta_{A},\theta_{B},\theta_{H}$ are unidentifiable as no coalescent events can occur in those populations given one sequence per species per locus. Population size $\theta_{S}$ is identifiable as it is possible for both sequences *a* and *b* to be traced back to population *S*. Nevertheless, one expects the information concerning $\theta_{S}$ to be weak in datasets of two sequences per locus. In methods c and d, $\theta_{S}$ and φ are estimated as free parameters. Application of the misspecified MSci model (to data generated under the IM model) led to unreasonably large estimates of $\theta_{S}$ (as large as 0.5 mutations per site), and the poor estimates of $\theta_{S}$ caused φ to be poorly estimated as well. This is due partly to our use of one sequence per species per locus and partly to the confounding effects between $\theta_{S}$ and φ. We discuss both effects later when we describe the simulation results. Here we focus on methods a, b, and e, in which $\theta_{S}$ is fixed at the true value $\theta_{0}$ (in methods a and b) or constrained to be equal to $\theta_{R}$ (in method e).

In the IM model, migration occurs throughout the time interval $\left(0,\tau_{R}\right)$, at the rate of $M_{AB}$ migrants per generation (fig. 1*a*). When such data are analyzed under the introgression model, a simple expectation might be that the introgression time $\tau_{S}$ should be the average $\tau_{R}/2$, whereas the introgression probability might be given by the expected proportion $\varphi_{0}$ of equation (10). However, as we show below, this expectation is too simplistic.

First, we discuss the introgression time $\tau_{S}$, assuming the true coalescent time (or n=∞). Given the data-generating IM model, there is a strictly positive probability for 0<t<ϵ for any small constant ϵ>0 (supplementary fig. S1, Supplementary Material online). In other words, there must exist loci at which *t* is arbitrarily close to 0. In the MSci model, sequences *a* and *b* cannot coalesce until they are in the same population *S*, so that $\tau_{S}<t$. When the MSci model is fitted to data generated under the IM model, ${\hat{\tau }}_{S}$ is dominated by the minimum rather than the average coalescent time, and ${\hat{\tau }}_{S}\to \tau_{S}^{*}=0$ when the number of loci L→∞ (and when the true coalescent time *t* is known). Even though migration occurs throughout the time interval $\left(0,\tau_{R}\right)$, the MSci model has to lump all migration events to one time point, $\tau_{S}^{*}=0$ (fig. 2*b*and*e*).

With finite sequences (n<∞), *t* is not observed and is reflected in the number of mutations (*x*). Whatever the true *t*, there is a positive probability of observing no mutations between the two sequences, so that an absence of mutations (x=0) is not strong evidence for t=0. The MLE $\tau_{S}^{*}$ reflects not only the minimum coalescent time, but also the whole distribution (supplementary fig. S1, Supplementary Material online). Thus $\tau_{S}^{*}>0$, different from the case where the coalescent time is known without error (n=∞). Nevertheless, one expects $\tau_{S}^{*}$ to be closer to 0 than to $\tau_{R}$, especially if the number of sites is large. Indeed in our calculations, $\tau_{S}^{*}\ll \frac{1}{2}\tau_{R}$ (fig. 2*b*and*e*).

Next we consider the introgression probability φ and again focus on methods a, b, and e (fig. 2). The estimate $\varphi^{*}$ increases nearly linearly when $M_{AB}$ is small ($<\frac{1}{4}$, say) but tails off at large $M_{AB}$. All estimates are smaller than $\varphi_{0}$ of equation (10) but they are close at low rates (with $M_{AB}<\frac{1}{4}$ and $\varphi <\frac{1}{4}$, say) (fig. 2*a*, *b*and*e*). We defer to a later section a detailed discussion of the estimation of φ, contrasting the IM, IIM, and SC models.

Finally the estimated divergence time between the two species ($\tau_{R}$) matched the true values at low migration rates but was underestimated at high migration rates, with the ancestral population size $\theta_{R}$ overestimated (fig. 2). It may be tempting to interpret the underestimation of $\tau_{R}$ (and overestimation of $\theta_{R}$) by the MSci model as being due to the difficulty of distinguishing complete isolation with recent species divergence from introgression or of distinguishing migration and coalescent events close to species divergence from ancestral polymorphism. However, this does not appear to be a correct interpretation.

We examined the true and fitted distributions of the coalescent time (supplementary fig. S1, Supplementary Material online). If there is no migration ($M_{AB}=0$), the MSci model (with φ=0) will be correct, and the parameter estimates will converge to the true values, with a perfect fit to the density $f_{m}(t)$. At low migration rates ($M_{AB}\leq 0.1$, say), the MSci model fits the density $f_{m}(t)$ very well, with the discontinuity point in the true and fitting distributions coinciding: $\tau_{R}^{*}=\tau_{R}^{m}$. At the intermediate rate of $M_{AB}=1$, the species divergence time $\tau_{R}$ is still correctly estimated even though the fit to the density is poor (supplementary fig. S1, Supplementary Material online). At high rates (with $M_{AB}\geq 1.4$, say), the true density has a mode in the interval $\left(0,\tau_{R}^{m}\right)$, dropping off at $\tau_{R}^{m}$. The best fitting density starts from 0, with an exponential decay, and has a discontinuity point at $\tau_{R}$ with again an exponential decay. This best-fitting density is a poor fit, and the discontinuity point $\tau_{R}^{*}$ is moved to smaller values as an attempt to accommodate the migration and coalescent events in the middle of the interval ($0,\tau_{R}^{m}$) to improve the fit (judged by the KL divergence). Thus $\tau_{R}$ is underestimated ($\tau_{R}^{*}<\tau_{R}^{m}$). As a result, the population size parameter $\theta_{R}$ is overestimated, as those two parameters tend to be strongly negatively correlated (e.g., Burgess and Yang 2008). In other words, the intermediate coalescent times in the interval (0, $\tau_{R}$), which occur at a large proportion of loci, are accommodated or misinterpreted by the MSci model using a smaller $\tau_{R}$ and larger $\theta_{R}$. Coalescent times in the range $\tau_{R}^{*}<t<\tau_{R}^{m}$, which represent true migration events, are misinterpreted as coalescent events in species *R*, and $\varphi^{*}$ is much less than $\varphi_{0}$ (eq. 10).

#### Asymptotic Results under the IIM Model

When data are generated under the IIM model (fig. 1*b*) and analyzed under the MSci model (fig. 1*d*), the results (supplementary figs. S2 and S3, Supplementary Material online) show similar patterns to those under the IM model discussed above. Similarly, $\theta_{S}$ is difficult to estimate using two sequences per locus in methods c and d, and the poor estimates of $\theta_{S}$ affects the estimation of φ. Thus we focus on methods a, b, and e, in which $\theta_{S}$ is fixed or constrained, and on the introgression time and introgression probability.

In the IIM model, migration events occur throughout the time interval $\left(\tau_{T},\tau_{R}\right)$ (fig. 1*b*), but the estimate of the introgression time is dominated or influenced by the minimum coalescent time, so that $\tau_{S}^{*}=\tau_{T}$ when n=∞, and $\tau_{S}^{*}>\tau_{T}$ when *n* is finite. In the latter case, $\tau_{S}^{*}$ is much closer to $\tau_{T}$ than to $\tau_{R}$ (supplementary fig. S2, Supplementary Material online).

The introgression probability $\varphi^{*}$ grew almost linearly with $M_{AB}$ when $M_{AB}$ was small (with $M_{AB}\leq 0.2$, say), and this estimate was close to the expectation $\varphi_{0}$ of equation (10) (supplementary fig. S2*a*,*b*and*e*, Supplementary Material online). At high migration rates, equation (10) gave a serious overestimate. This “bias” in φ at high migration rates was accompanied by a reduction in $\tau_{R}$ and overestimation of $\theta_{R}$. This can similarly be explained by the attempt of the MSci model to accommodate the coalescent times in the middle of the time interval ($\tau_{T},\tau_{R}$) (supplementary fig. S3, Supplementary Material online).

#### Asymptotic Results under the SC Model

Under the SC model, there is initially complete isolation after species divergence but the two species come into contact at time $\tau_{T}$, with ongoing gene flow ever since (fig. 1*c*). The best-fitting parameter values under the MSci model ($\Theta_{i}^{*}$), for data of two sequences per locus, are shown in supplementary figure S4, Supplementary Material online, with fitted densities of coalescent time *t* shown in supplementary figure S5, Supplementary Material online.

The results show patterns similar to those under the IM and IIM models discussed above. The species divergence time under the MSci model $\tau_{R}^{*}=\tau_{R}^{\left(\mathrm{sc}\right)}$ when the migration rate $M_{AB}$ is small but drops at very high rates (with $M_{AB}>2$). The introgression time is dominated by the minimum coalescent time, so that $\tau_{S}^{*}=0$ when n=∞, and $\tau_{S}^{*}$ is much closer to 0 than to $\tau_{T}$ when *n* is finite (supplementary fig. S4, Supplementary Material online). Note that in the true model migration occurs throughout the time interval $\left(0,\tau_{T}\right)$.

The introgression probability $\varphi^{*}$ grew almost linearly with the migration rate $M_{AB}$ when $M_{AB}$ was small (with $M_{AB}\leq \frac{1}{4}$, say), and was close to the expectation $\varphi_{0}$ (eq. 10) when $M_{AB}<2$ (supplementary fig. S4*a*,*b*and*e*, Supplementary Material online). At very high rates ($M_{AB}>2$), $\varphi^{*}$ was much smaller than $\varphi_{0}$, and this ‘bias’ was accompanied by an underestimation of $\tau_{R}$ and overestimation of $\theta_{R}$. Similarly to the IM and IIM models discussed above, this is due to the attempt of the MSci model to accommodate the coalescent times in the middle of the interval ($0,\tau_{T}$) (supplementary fig. S5, Supplementary Material online).

#### The Amount of Gene Flow under the IM, IIM, and SC Models

Although the expected total amount of gene flow measured by $\varphi_{0}$ (eq. 10) is the same under the IM, IIM, and SC models of figure 1*a*–*c*, the estimates under the MSci model differ, as summarized in figure 1*e*.

At low migration rates, $\tau_{R},\theta_{S}$, and $\theta_{R}$ in the MSci model are nearly accurately estimated to match those in the true model (figs. 2, supplementary figures S2 and S4, Supplementary Material online). Consider the case of infinitely long sequences with known coalescent time. Let $\tau_{R}^{*}=\tau_{R}^{m}$, $\theta_{R}^{*}=\theta_{R}^{m}$, and let the introgression time be $\tau_{S}^{*}=0$ for the IM and SC model, and $\tau_{S}^{*}=\tau_{T}$ for the IIM model. We also match the probability density of coalescent time *t*, with $f_{i}(t)=f_{m}(t)$, for $t>\tau_{R}$. With those simplifying assumptions, $\varphi^{*}$ that minimizes the KL divergence (eq. 9) can be derived as

(11) $$\begin{array}{cc} \varphi_{\left(\mathrm{IM}\right)}^{*} & \;\;\;\;\;\approx \frac{\varphi_{0}-\frac{w\theta_{A}}{2}(1-e^{-\frac{2}{\theta_{A}}\tau_{R}})}{\left(1-\frac{w\theta_{A}}{2})(1-e^{-\frac{2}{\theta_{A}}\tau_{R}}\right)},\\ \varphi_{\left(\mathrm{IIM}\right)}^{*} & \;\;\;\;\;\approx \frac{\varphi_{0}-\frac{w\theta_{A}}{2}(1-e^{-\frac{2}{\theta_{A}}(\tau_{R}-\tau_{T})})}{\left(1-\frac{w\theta_{A}}{2})(1-e^{-\frac{2}{\theta_{A}}(\tau_{R}-\tau_{T})}\right)},\\ \varphi_{\left(\mathrm{SC}\right)}^{*} & \;\;\;\;\;\approx \frac{\varphi_{0}-\frac{w\theta_{A}}{2-w\theta_{A}}(e^{-w\tau_{T}}-e^{-\frac{2}{\theta_{A}}\tau_{T}})e^{-\frac{2}{\theta_{A}}(\tau_{R}-\tau_{T})}}{1-e^{-\frac{2}{\theta_{A}}\tau_{R}}}. \end{array}$$

At low migration rates, equation (11) provides accurate numerical results (methods a, b, e in figs. 2, supplementary figures S2 and S4, Supplementary Material online). From equation (11), we have

(12) $$\varphi_{0}>\varphi_{\left(\mathrm{SC}\right)}^{*}>\varphi_{\left(\mathrm{IIM}\right)}^{*}=\varphi_{\left(\mathrm{IM}\right)}^{*}.$$

In other words, recent gene flow (as in SC) is easier to recover by the MSci model than ancient gene flow (as in IM or IIM). Note that $\varphi_{\left(\mathrm{IIM}\right)}^{*}=\varphi_{\left(\mathrm{IM}\right)}^{*}$ holds only when one sequence is sampled per species; as there is no coalescent over $\left(0,\tau_{T}\right)$, IIM is essentially the same model as IM with a time shift (fig. 1). This will not be the case when multiple sequences per species are sampled or when the sequence length is finite.

#### Simulation Results

**Fig. 3.**
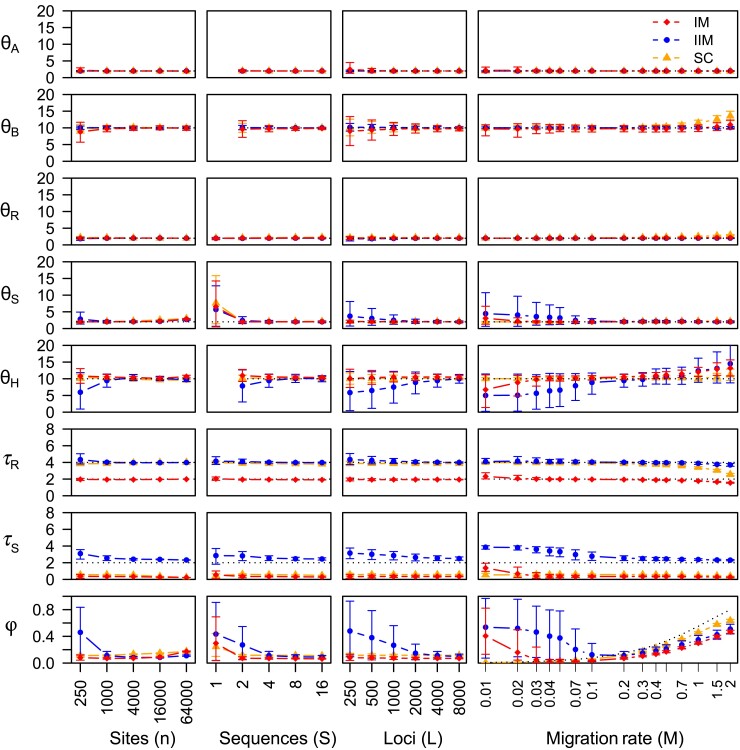
Average posterior means and 95% HPD CIs for parameters in the MSci model of figure 1*d* over 30 replicate datasets simulated under the migration (IM, IIM, and SC) models of figure 1*a*–*c*, plotted against the number of sites per sequence (*n*), the number of sequences per species (*S*), the number of loci (*L*), and the migration rate ($M_{AB}$). Parameters in the migration model are given in the legend to figure 1. In the standard setting, each dataset consists of L=4,000 loci, with S=4 sequences per species at each locus and n=1,000 sites per sequence, and the migration rate was $M_{AB}=0.2$ individuals per generation. In the four sets of simulations, one of the factors (n,S,L,M) varies whereas the others are fixed. When S=1, population sizes $\theta_{A}$, $\theta_{B}$, and $\theta_{H}$ are unidentifiable. Estimates of τ and θ parameters are multiplied by $10^{3}$. Dotted lines indicate true values of identifiable parameters, except in the plot of φ against $M_{AB}$, where it represents $\varphi_{0}$ of equation (10), (which is identical for the IM, IIM, and SC models of fig. 1). Note that the *n*, *S*, *L*, and $M_{AB}$ axes are all on the logarithmic scale.

As our asymptotic theory was limited to a single sequence per species per locus, we used simulation to verify and augment our analytical calculations above. We simulated data under the IM, IIM, or SC models of figure 1*a*–*c*, using the same parameter values as above, and analyzed them using Bpp under the MSci model (fig. 1*d*). The JC mutation model (Jukes and Cantor 1969) was assumed. In the basic setting, we used S=4 sequences per species per locus, n=1,000 sites per sequence, and L=4,000 loci in each dataset, with the migration rate $M_{AB}=0.2$. We varied $n,S,L,M_{AB}$ to examine their effects. With multiple sequences per species (S>1), all eight parameters of the MSci model, $\Theta_{i}=(\varphi ,\theta_{A},\theta_{B},\theta_{R},\theta_{S},\theta_{H},\tau_{R},\tau_{S})$ (fig. 1*d*) are identifiable (Yang and Flouri 2022). The results are summarized in figure 3. We note a few common features first. In nearly all cases, population sizes for extant species ($\theta_{A},\theta_{B}$) were very well estimated, with posterior means close to the true values and with very narrow highest-probability-density (HPD) credibility intervals (CIs). The exception was parameter $\theta_{B}$ under the IM model (note that *B* is the species receiving immigrants), which was less well estimated when the dataset was small and had either short sequences (n=250) or few loci (L≤500), or when the migration rate was very high. The poorer estimation of $\theta_{B}$ appeared to be related to the underestimation of φ and $\tau_{R}$; see below. The population size for the common ancestor $\theta_{R}$ was mostly well estimated, although overestimated at very high migration rates. Population sizes for the ancestral species ($\theta_{S},\theta_{H}$) are harder to estimate; indeed they had larger CIs and were influenced by misspecification of the model of gene flow. As expected from the asymptotic results, $\tau_{R}$ was very well estimated, except at very high migration rates, in which case $\tau_{R}$ was underestimated (and $\theta_{R}$ overestimated).

Next we examine the effects of $n,S,L,M_{AB}$ in turn. First, the number of sites (*n*) had a relatively small impact on MSci parameters, when other factors were fixed (at the basic setting of S=4,L=4,000, and $M_{AB}=0.2$). When n=250, CIs for parameters such as the introgression time and probability ($\tau_{S}$ and φ) were wide. When n≥1,000, the CIs were much smaller for all parameters. The introgression time ${\hat{\tau }}_{S}$ decreased slightly as the sequence became longer. This is consistent with the asymptotic analysis, which suggests that $\tau_{S}^{*}$ is dominated by the smallest coalescent time or sequence divergence and should be 0 for the IM and SC models or $\tau_{T}$ for the IIM model (when n→∞) (figs. 2, supplementary figures S2 and S4, Supplementary Material online). Similarly, for the IM and SC models, $\hat{\varphi }$ increased with the increase of *n* when $M_{AB}$ was low, as observed in the asymptotic analysis. Under the IIM model, small datasets with short sequences (n=250) produced very uncertain estimates of φ and $\theta_{H}$ (and, to a lesser extent, $\tau_{S}$ and $\theta_{S}$). The two parameters are nearly confounded; this is discussed below when we examine the impact of the migration rate ($M_{AB}$).

Second, we varied the number of sequences per species (*S*). When one sequence per species is in the data (S=1), only five parameters in the MSci model (fig. 1*d*) are identifiable: $\theta_{R},\theta_{S},\tau_{R},\tau_{S},\varphi$). When multiple sequences were sampled per species, all eight parameters are identifiable. They were well estimated when the dataset was large (say, with S≥2 for IM and SC or S≥4 for IIM). Even with S=4 sequences per species, estimates of φ from data generated under the IIM model involved wide CIs, with $\tau_{S}$ being close to $\tau_{R}$, and $\theta_{S}$ and $\theta_{H}$ being very imprecise as well (fig. 3). This is due to the semi-unidentifiability or the confounding effects of the parameters, and will be discussed below. Here we note that the problem disappeared and all parameter estimates were well-behaved in large datasets when many sequences were sampled (S≥2 for IM and SC or S≥4 for IIM; fig. 3).

Third, we examined the impact of the number of loci (*L*). The IIM model was hard to fit in small datasets with a small number of loci (L≤1,000), generating large CIs for parameters φ and $\theta_{H}$. This is the same pattern as in the case of short loci (n=250) or few sequences (S≤2), discussed above. In large datasets, the parameters were well estimated. Note that the number of loci *L* is the sample size in the statistical model as data at different loci are independently and identically distributed. Theory predicts that in large datasets the variance should be proportional to 1/L (see O’Hagan and Forster 2004 for the case of correctly specified models and Yang and Zhu 2018 for the case of misspecified models), and thus the CI should decrease at the rate of $L^{-1/2}$. This prediction held for parameters that were well estimated (fig. 3). As discussed earlier, the introgression time $\tau_{S}$ is dominated by the smallest coalescent time or smallest sequence divergence. Thus increasing the number of loci led to a decrease in the estimated introgression time, and the trend was in particular apparent for the IIM model (under which ${\hat{\tau }}_{S}\to \tau_{T}$ when L→∞ if n=∞). In all cases, the estimated introgression time (${\hat{\tau }}_{S}$) was closer to the more recent end of the time interval for gene flow than to the midpoint, that is, ${\hat{\tau }}_{S}<\tau_{R}/2$ for IM, ${\hat{\tau }}_{S}<\left(\tau_{R}+\tau_{T}\right)/2$ for IIM, and ${\hat{\tau }}_{S}<\tau_{T}/2$ for SC (see fig. 1*a*–*c*).

Finally, we evaluated the impact of the migration rate ($M_{AB}$) (fig. 3). Under the IM model, there is a near linear relationship between the introgression probability φ and $M_{AB}$ at low rates. The amount of gene flow estimated under the MSci model is less than the true amount expected under the IM model ($\varphi_{0}$ of eq. 10) but the two were close at low rates (with $M_{AB}<0.1$, say). At very high rates (with $M_{AB}>1.0$, say), divergence time $\tau_{R}$ was increasingly underestimated and the population size $\theta_{R}$ was overestimated. These patterns are the same as observed in the asymptotic analysis of infinite data (L=∞), and are due to the attempt of the MSci model to accommodate intermediate coalescent times in the data, as discussed earlier (see figs. 2, supplementary figures S2 and S4, Supplementary Material online).

Under the IIM model, $\hat{\varphi }$ involved very large uncertainties at low rates ($M_{AB}<0.04$, say), with $\theta_{S},\tau_{S}$, and $\theta_{H}$ affected as well. Given the small $M_{AB}$, why did $\hat{\varphi }$ not converge to ∼0 with narrow CIs? Note that if $M_{AB}=0$ in the IIM model, the MSC model with no gene flow or MSci with φ=0 will be the correct model. Similarly in figure 3 where $M_{AB}=0.2$ was fixed, wide CIs for those parameters were observed in small datasets with short loci (n≤250), few sequences (S≤2), or few loci (L≤1,000), as noted above. Also in the asymptotic analysis (with L=∞), we noted that $\theta_{S}^{*}$ and $\varphi^{*}$ were grossly wrong but had no sampling errors because the data size was L=∞ (fig. 2; supplementary figures S2 and S4, Supplementary Material online, methods c, d). We suggest that all those results are due to the near unidentifiability of parameters in the MSci model (in particular, $\theta_{S}$ and φ); in other words, the parameters are confounded.

If $M_{AB}=0$ in the MSC-M model, MSci with φ=0 will be the correct model, but φ>0 with a large appropriately adjusted $\theta_{S}$ may provide a very good fit to the data (of two sequences per locus). When $M_{AB}$ is small but nonzero, the MSci model will never achieve a perfect fit, and a large φ with appropriately adjusted $\theta_{S}$ may provide a better fit than a small φ. Thus in infinite data (L=∞), we may get grossly wrong estimates with no uncertainty (supplementary figure S2, Supplementary Material online, methods c, d). In finite datasets (L<∞), there will be a ridge in the posterior surface involving φ and $\theta_{S}$, leading to wide CIs for those parameters, influenced by both model misspecification and the prior (fig. 3). Including multiple samples from the same species (S>1) is useful for improving the information content in the data, but strong correlation between $\hat{\varphi }$ and ${\hat{\theta }}_{S}$ may be expected nevertheless. In this regard, the large uncertainties in posterior estimates of parameters may be useful as they help the investigator avoid incorrect inferences of a large φ when gene flow is minimal.

### Introgression Events Assigned to Wrong Branches

**Fig. 4.**
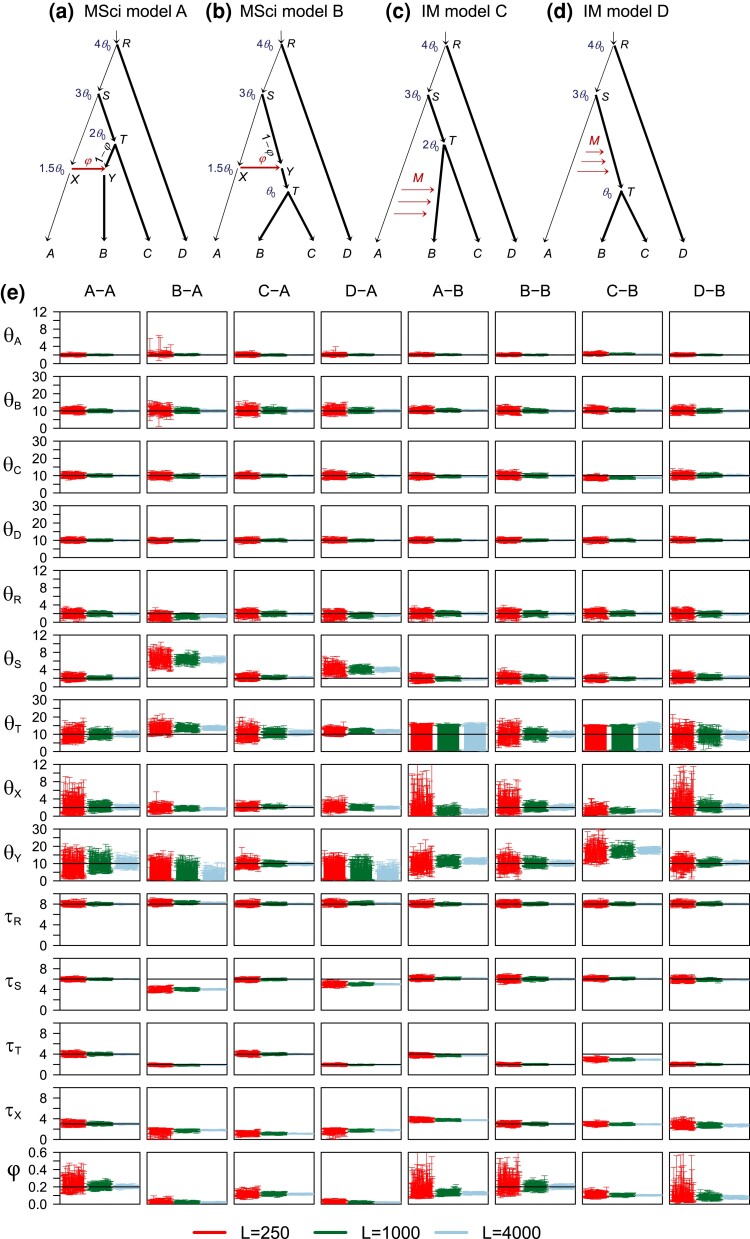
(*a*,*b*) Two introgression (MSci) models and (*c*,*d*) two migration (IM) models used in simulation. The thin branches have the population size $\theta_{0}=0.002$ and the thick branches have $\theta_{1}=0.01$. In MSci model A, the species divergence/introgression times are $\tau_{R}=4\theta_{0}$, $\tau_{S}=3\theta_{0}$, $\tau_{T}=2\theta_{0}$, and $\tau_{X}=\tau_{Y}=1.5\theta_{0}$. In MSci model B, $\tau_{R}=4\theta_{0}$, $\tau_{S}=3\theta_{0}$, $\tau_{T}=\theta_{0}$, and $\tau_{X}=\tau_{Y}=1.5\theta_{0}$. Introgression probability is φ=0.2. In the IM model C, $\tau_{R}=4\theta_{0}$, $\tau_{S}=3\theta_{0}$, and $\tau_{T}=2\theta_{0}$, with migration occurring from species *A* to *B* over time period ($0,\tau_{T}$) at the rate M=0.1 migrants per generation. In the IM model D, $\tau_{R}=4\theta_{0}$, $\tau_{S}=3\theta_{0}$, and $\tau_{T}=\theta_{0}$, with migration from species *A* to *ST* over time period ($\tau_{T},\tau_{S}$) at the rate M=0.1. (*e*) The 95% HPD CIs for parameters in 100 replicate datasets of L=250, 1,000, and 4,000 loci. Column labels refer to the simulation model followed by the analysis model; for example, “B-A” means that data were simulated under model B and analyzed under model A. The values of θ and τ parameters are multiplied by $10^{3}$. Black solid line indicates the true value.

We conducted simulations to examine the bias in parameter estimates when the introgression event is assigned on either the parental or daughter branch of the lineage genuinely involved in introgression. The data were simulated under model trees A or B and analyzed under models A or B of figure 4*a*and*b*.

In the A-A and B-B settings (fig. 4*e*), the correct MSci model was assumed, and the performance of the method serves as a reference for comparison. Most parameters, including the species divergence times ($\tau_{R},\tau_{S},\tau_{T}$, and $\tau_{X}=\tau_{Y}$) and population sizes for extant species ($\theta_{A},\theta_{B},\theta_{C},\theta_{D}$), were well estimated. For well-estimated parameters, the CI width reduced by a half as the number of loci (*L*) quadrupled, as predicted by theory. Population sizes for ancestral species ($\theta_{R}$, $\theta_{S}$, $\theta_{T}$, $\theta_{X}$, and $\theta_{Y}$) were less well estimated, although performance improved with sample size: with L=4,000 loci, these parameters were well estimated. Introgression probability (φ) was well estimated, but thousands of loci were necessary to obtain precise estimates with narrow CIs under the standard settings used here (four sequences per species per locus and 500 sites per sequence).

In the other settings (fig. 4*e*), there was mismatch between the models used to simulate and to analyze data. We note that population sizes for extant species ($\theta_{A},\theta_{B},\theta_{C},\theta_{D}$) were well estimated, as was the age of the root ($\tau_{R}$). Performance for estimation of those parameters was very similar whether or not there was model misspecification (e.g., the A-B setting versus the B-B setting and C-A versus A-A). Below we focus on estimation of the other parameters.

**Fig. 5.**
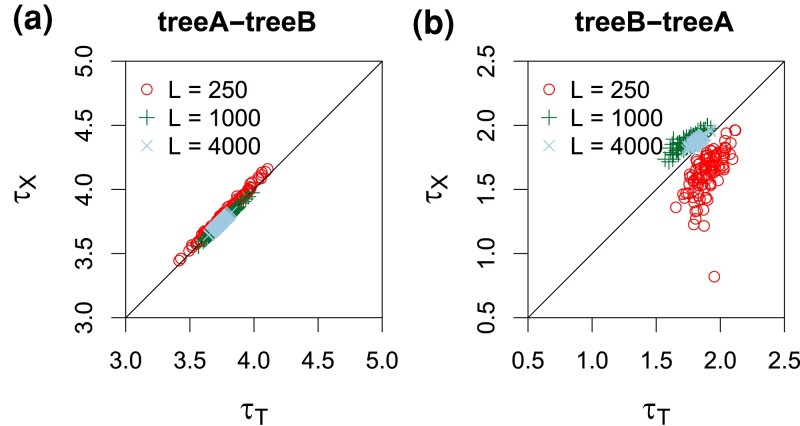
Posterior means of speciation/introgression times ($\times 10^{-3}$) when the introgression event is assigned to a wrong branch. In (*a*) tree A-tree B, data were simulated using species tree A (fig. 4*a*), with introgression from species *A* to *B*, but are analyzed assuming tree B, with introgression assigned incorrectly to the parental branch *ST* (so that $\tau_{X}>\tau_{T}$). In (*b*) tree B-tree A, data were simulated under tree B (fig. 4*b*) and analyzed assuming tree A, with introgression assigned to the daughter branch *B* (with $\tau_{X}<\tau_{T}$). For each number of loci (L=250, 1,000, 4,000), 100 replicate datasets were generated and analyzed. These correspond to the A-B and B-A settings of figure 4*e*, where estimates of other parameters are shown.

In the A-B setting (fig. 4*e*), data were simulated under model A with A→B introgression (fig. 4*a*) but analyzed under model B with introgression incorrectly assigned to the parental branch *ST*. Ancestral population sizes $\theta_{R}$ and $\theta_{S}$ were well estimated, similar to the B-B setting. Divergence times $\tau_{R}$ and $\tau_{S}$ were well estimated, but ${\hat{\tau }}_{T}$ and ${\hat{\tau }}_{X}$ were stuck together. We expect ${\hat{\tau }}_{T}^{\left(B\right)}$ to be mostly determined by the smallest sequence divergence ($t_{bc}$) between *B* and *C*, which should be close to $\tau_{T}^{\left(A\right)}=2\theta_{0}=0.004$. Here, we use the superscript to indicate the model in which the parameter is defined. In the fitting model B, the introgression time ${\hat{\tau }}_{X}^{\left(B\right)}$ (which is $>{\hat{\tau }}_{T}^{\left(B\right)}$) should reflect the smallest sequence divergence $t_{ab}$, whereas in the true model A, $t_{ab}$ is mostly determined by $\tau_{X}$ (which is $<\tau_{T}$). Thus misidentification of the introgression lineage caused ${\hat{\tau }}_{X}^{\left(B\right)}$ to be stuck at ${\hat{\tau }}_{T}^{\left(B\right)}$ (fig. 5*a*). There was virtually no information for $\theta_{T}$ as the population was estimated to have near-zero time duration with no chance for coalescence. The introgression probability was seriously underestimated, converging to $\varphi_{A-B}^{*}\approx 0.12$ when the number of loci *L* increases (table 1), whereas the true value was 0.2. This smaller estimate of introgression probability is explained by the distribution of coalescent times between species in the true and fitting models (supplementary fig. S6, Supplementary Material online, true model A). Under the true model A, sequences from *A* and *B* are more similar than those between *A* and *C* due to the A→B introgression, with an excess of small coalescence time $t_{ab}$. Under the analysis model B, $t_{ab}$ and $t_{ac}$ have the same distribution. Thus the true model predicts an excess of small $t_{ab}$, whereas the fitting model predicts an excess of small $t_{ac}$, and having a smaller φ in the fitting model helps to reduce the discrepancy.

**Table 1.** Average Posterior Means and 95% HPD Intervals (in parentheses) for Introgression Time ($\tau_{X}$, $\times 10^{-3}$) and Introgression Probability ($\varphi_{X}$) in the Simulations.

Analysis	$\tau_{X}$			φ		
	L=250	L=1000	L=4000	L=250	L=1000	L=4000
Figure 4 A-A	3.06 (2.63, 3.49)	3.02 (2.80, 3.24)	3.00 (2.89, 3.11)	0.23 (0.16, 0.32)	0.21 (0.17, 0.24)	0.20 (0.19, 0.22)
Figure 4 B-A	1.62 (0.95, 2.05)	1.77 (1.54, 1.96)	1.82 (1.72, 1.91)	0.02 (0.00, 0.04)	0.02 (0.01, 0.03)	0.02 (0.02, 0.03)
Figure 4 C-A	1.12 (0.83, 1.40)	1.11 (0.97, 1.25)	1.11 (1.04, 1.18)	0.12 (0.09, 0.15)	0.12 (0.10, 0.13)	0.12 (0.11, 0.12)
Figure 4 D-A	1.69 (1.18, 2.07)	1.80 (1.58, 1.97)	1.86 (1.76, 1.94)	0.02 (0.01, 0.04)	0.02 (0.01, 0.03)	0.02 (0.02, 0.03)
Figure 4 A-B	3.82 (3.53, 4.11)	3.75 (3.61, 3.90)	3.73 (3.66, 3.80)	0.18 (0.09, 0.28)	0.13 (0.11, 0.16)	0.12 (0.11, 0.14)
Figure 4 B-B	2.98 (2.61, 3.35)	2.99 (2.80, 3.18)	3.00 (2.91, 3.10)	0.23 (0.14, 0.34)	0.20 (0.17, 0.24)	0.20 (0.18, 0.22)
Figure 4 C-B	2.98 (2.72, 3.24)	2.93 (2.80, 3.06)	2.91 (2.85, 2.98)	0.11 (0.08, 0.14)	0.10 (0.09, 0.12)	0.10 (0.10, 0.11)
Figure 4 D-B	2.83 (2.28, 3.38)	2.71 (2.42, 3.00)	2.73 (2.59, 2.87)	0.11 (0.04, 0.20)	0.08 (0.05, 0.10)	0.08 (0.07, 0.09)
Figure 6 IIM	3.40 (2.38, 4.36)	2.93 (2.42, 3.43)	2.83 (2.58, 3.08)	0.24 (0.04, 0.53)	0.10 (0.05, 0.16)	0.08 (0.06, 0.10)
Figure 7	2.81 (2.41, 3.22)	2.80 (2.60, 3.01)	2.79 (2.68, 2.89)	0.23 (0.16, 0.31)	0.21 (0.18, 0.25)	0.21 (0.19, 0.22)
Figure 8	3.12 (1.93, 4.07)	3.05 (2.42, 3.68)	2.98 (2.73, 3.23)	0.03 (0.01, 0.06)	0.03 (0.01, 0.04)	0.02 (0.02, 0.03)

In the B-A setting (fig. 4*e*), the simulation model (MSci model B of fig. 4*b*) assumes introgression involving the ancestral branch *ST* but the analysis model (model A) assigned introgression to the daughter branch *TB*. Posterior means and CIs for divergence times $\tau_{R}$ and $\tau_{T}$ were similar to those in the A-A setting. Note that ${\hat{\tau }}_{T}^{\left(A\right)}$ should be mostly determined by the smallest sequence divergence ($t_{bc}$) between *B* and *C*, and given that this is $\tau_{T}^{\left(B\right)}=2\theta_{0}=0.002$, ${\hat{\tau }}_{T}^{\left(A\right)}$ was well estimated, unaffected by mis-assigned introgression event. Although the true introgression time $\tau_{X}$ was 0.003, it was forced to be less than $\tau_{T}$ by the analysis model A. As the number of loci increases, ${\hat{\tau }}_{X}^{\left(A\right)}$ became stuck at ${\hat{\tau }}_{T}^{\left(A\right)}$ (fig. 5*b*). However, ${\hat{\tau }}_{S}^{\left(A\right)}$ was seriously underestimated. This may be explained as follows. In the analysis model A, ${\hat{\tau }}_{S}^{\left(A\right)}$ was mostly determined by the shortest sequence distance between *A* and *C*. In the true model B, this should be close to $\tau_{X}^{\left(B\right)}=1.5\theta_{0}=0.003$, due to introgression. With mutational fluctuations in the sequences, one can expect ${\hat{\tau }}_{S}^{\left(A\right)}$ to lie between $\left(\tau_{X}^{\left(B\right)},\tau_{S}^{\left(B\right)})=(1.5\theta_{0},3\theta_{0}\right)$, but closer to $\tau_{X}^{\left(B\right)}$ in large datasets with many sites and/or many loci. Population sizes ${\hat{\theta }}_{S}^{\left(A\right)}$ and ${\hat{\theta }}_{Y}^{\left(A\right)}$ were affected by the mis-assigned introgression events as well, as those populations are close to the introgression branches. In particular, ${\hat{\theta }}_{Y}^{\left(A\right)}$ was very imprecise as branch *YT* was very short, and ${\hat{\theta }}_{S}^{\left(A\right)}$ was overestimated because ${\hat{\tau }}_{S}^{\left(A\right)}$ was seriously underestimated (as those two parameters are negatively correlated). Finally, the introgression probability (φ) was underestimated, apparently converging to $\varphi_{B-A}^{*}\approx 0.02$ when the number of loci *L* increased (table 1), whereas the true value was 0.2. This greatly reduced introgression probability appeared to reflect the very poor fit of the misspecified model A to data generated under model B (see the large differences between the true and fitting distributions of coalescent times in supplementary fig. S6, Supplementary Material online, second row). As ${\hat{\tau }}_{X}^{\left(A\right)}$ and ${\hat{\tau }}_{S}^{\left(A\right)}$ are seriously underestimated, an excess of small coalescent times ($t_{ab},t_{ac}$) is expected in the fitting model A but does not appear in the data, so that having a smaller φ improves the fit.

In summary, assigning introgression events to a wrong parental or daughter branch led to biased estimates of introgression times (causing the introgression events to collapse onto speciation events) and to seriously underestimated introgression probabilities.

### Continuous Migration versus Episodic Introgression

In this set of simulations, we generated data under the IM models C and D of figure 4*c*and*d* and analyzed them under the MSci models A and B, with the mode of gene flow misspecified and with gene flow assigned to either the correct branch or a wrong branch on the species tree.

In the C-A and D-B settings (fig. 4*e*), gene flow occurred continuously but the data were analyzed under the MSci model assuming introgression at a time point. The mode of gene flow was misspecified, but the lineages involved were correctly identified. In the C-A setting, gene flow was between non-sister species, whereas in the D-B setting it was between sister species. Speciation times ($\tau_{R}$, $\tau_{S}$, $\tau_{T}$) and population sizes (θ) were well estimated, similar to the A-A setting. Surprisingly ancestral population sizes $\theta_{T},\theta_{X},\theta_{Y}$ appeared to be even better estimated, with narrower CIs, in the C-A setting than in A-A. Speciation times and population sizes were extremely similar between settings D-B and B-B. Those results were consistent with the results for the case of two species (fig. 3), which showed that at low migration rates, species divergence times and population sizes were well estimated under the MSci model when the data were generated under the IM model.

In the C-A setting, the estimated introgression time ${\hat{\tau }}_{X}^{\left(A\right)}$ appeared to converge (when *L* increased) to 0.0011, much more recent than the average time of gene flow ($\tau_{T}^{\left(C\right)}/2=0.002$), and the introgression probability ${\hat{\varphi }}_{C-A}$ appeared to converge to $\varphi_{C-A}^{*}=0.12$ (table 1), smaller than the expected proportion of total migrants: $\varphi_{0}=1-e^{-4M_{AB}\tau_{T}^{\left(C\right)}/\theta_{B}^{\left(C\right)}}=0.148$. As discussed earlier for the case of two species, the limiting value for ${\hat{\tau }}_{X}^{\left(A\right)}$ was nonzero, as the sequence length is finite, and the MLE ${\hat{\varphi }}_{C-A}$ slightly underestimated the true amount of gene flow. In the D-B setting, the introgression time ${\hat{\tau }}_{X}^{\left(B\right)}$ appeared to converge to 0.0027, larger than $\tau_{T}^{\left(D\right)}=0.002$ but much smaller than the average time of gene flow, $\frac{1}{2}(\tau_{S}^{\left(D\right)}+\tau_{T}^{\left(D\right)})=0.004$, and the introgression probability ${\hat{\varphi }}_{D-B}$ appeared to converge to $\varphi_{D-B}^{*}=0.08$ (table 1), much smaller than $\varphi_{0}=0.148$ from equation (10). In both the C-A and D-B settings, the estimated introgression time was within the time interval of gene flow, but closer to the time when gene flow stopped, whereas the amount of gene flow was underestimated ($\varphi_{C-A}^{*}<\varphi_{0}$, $\varphi_{D-B}^{*}<\varphi_{0}$). Moreover, we have $\varphi_{D-B}^{*}<\varphi_{C-A}^{*}$. These patterns are consistent with our analysis of the two-species case at low migration rates (eq. 12, fig. 3), which suggested that gene flow after a period of isolation (the SC model) is easier to recover than gene flow that starts at speciation but stops some time afterwards (the IIM model).

In the C-B and D-A settings (fig. 4*e*), the mode of gene flow was misspecified and furthermore gene flow was assigned onto the wrong branch of the species tree. In the C-B setting, divergence time $\tau_{T}$ was underestimated slightly, due to gene flow assigned to the wrong branch, as observed in the A-B setting. Ancestral population sizes $\theta_{T}$ and $\theta_{Y}$ were affected by gene flow, similar to the A-B setting. Model B forces $\tau_{X}>\tau_{T}$. Thus we expect ${\hat{\tau }}_{X}^{\left(B\right)}$ and ${\hat{\tau }}_{T}^{\left(B\right)}$ to get stuck together, with both being smaller than $\tau_{T}^{\left(C\right)}=2\theta_{0}=0.004$; as the number of loci *L* increased, ${\hat{\tau }}_{X}^{\left(B\right)}$ appeared to converge to 0.0029, and ${\hat{\varphi }}_{C-B}$ to $\varphi_{C-B}^{*}=0.10$ (table 1).

In the D-A setting, the divergence time $\tau_{S}$ was underestimated, due to gene flow assigned to the wrong branch, similarly to the B-A setting. The ancestral population sizes $\theta_{R}$ and $\theta_{X}$ were well estimated as in the A-A setting, but $\theta_{T}$ had a slight positive bias. The ancestral population sizes $\theta_{S}$ and $\theta_{Y}$ were affected by the gene flow, similar to the B-A setting. The introgression time and probability ($\tau_{X}$ and φ) do not exist in the simulation model D. Model A forces $\tau_{X}<\tau_{T}$, so we expect ${\hat{\tau }}_{X}^{\left(A\right)}$ to be close to $\tau_{T}^{\left(D\right)}=\theta_{0}=0.002$; when the number of loci *L* increased, ${\hat{\tau }}_{X}^{\left(A\right)}$ appeared to converge to 0.00186, and ${\hat{\varphi }}_{D-A}$ to $\varphi_{D-A}^{*}=0.02$ (table 1). Note that $\varphi_{0}>{\hat{\varphi }}_{C-B}>{\hat{\varphi }}_{D-A}$ with ${\hat{\varphi }}_{C-B}<{\hat{\varphi }}_{C-A}$ and ${\hat{\varphi }}_{D-A}<{\hat{\varphi }}_{D-B}$. Those results are consistent with our early results for fitting the MSci model to data generated under the migration model in the two-species case (eq. 12, fig. 3), and with the results for the A-B and B-A settings that assignment of gene flow to a wrong branch reduces the estimate of φ.

In summary, the estimated introgression probabilities, at 0.12, 0.08, 0.10, and 0.02 for the C-A, D-B, C-B, and D-A settings, respectively, even though the total amount of gene flow was the same in models C and D (table 1), suggest the following general patterns. First, the MSci model underestimates the total amount of gene flow if gene flow occurs continuously in every generation (i.e., ${\hat{\varphi }}_{C-A}<\varphi_{0},{\hat{\varphi }}_{D-B}<\varphi_{0}$), as discussed in our analysis of the two-species case. Second, assigning gene-flow events to wrong lineages led to serious underestimation of the amount of gene flow (i.e., ${\hat{\varphi }}_{C-B}<{\hat{\varphi }}_{C-A},{\hat{\varphi }}_{D-A}<{\hat{\varphi }}_{D-B}$). Third, recent gene flow in the data is more easily recovered (i.e., ${\hat{\varphi }}_{C-A}>{\hat{\varphi }}_{D-B},{\hat{\varphi }}_{C-B}>{\hat{\varphi }}_{D-A}$).

### Isolation with Initial Migration (IIM) Model

**Fig. 6.**
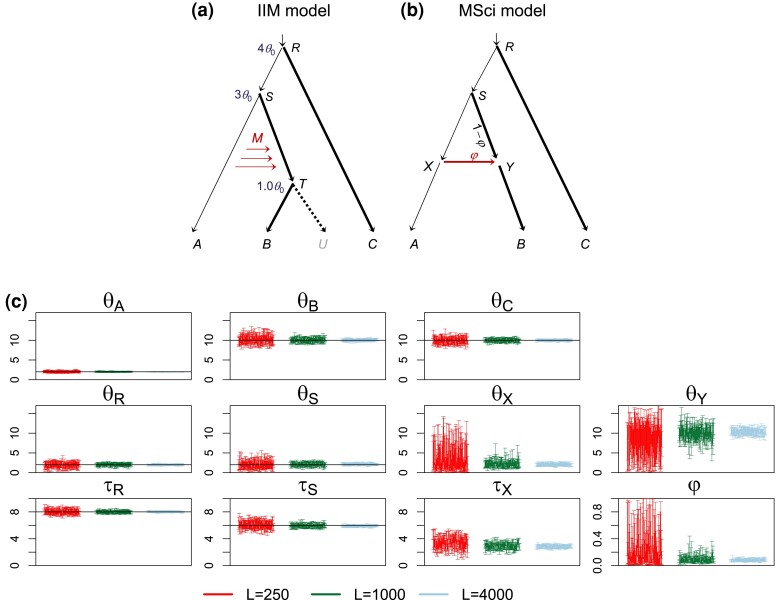
(*a*) An isolation-with-initial-migration (IIM) model used to simulate data. The parameter values used are $\theta_{0}=0.002$ for population sizes for the thin branches and $\theta_{1}=0.01$ for the thick branches, $\tau_{R}=4\theta_{0},\tau_{S}=3\theta_{0},\tau_{T}=\theta_{0}$ for species divergence times. The number of sequences is S=4, with the sequence length n=500. The migration rate is M=0.1. (*b*) The MSci model used to analyze the data. (*c*) The 95% HPD CIs for parameters, with black lines indicating the true values. Estimates of θ and τ are multiplied by $10^{3}$.

Next, we assessed the effects of taxon sampling when the mode of gene flow is misspecified. We used the IIM model for three species of figure 6*a* to simulate data and analyzed them under the MSci model of figure 6*b*. Species divergence times ($\tau_{R},\tau_{S}$) and population sizes ($\theta_{A},\theta_{B},\theta_{C},\theta_{R},\theta_{S}$, and even $\theta_{X}$ and $\theta_{Y}$) were well estimated. We expect the estimated introgression time ${\hat{\tau }}_{X}$ to converge to $\tau_{T}=\theta_{0}=0.002$ if the sequence length is infinite and to a higher limit for finite sequence length. In our simulation ${\hat{\tau }}_{X}\approx 0.00283$ at L=4,000 (table 1). The estimated introgression probability ($\hat{\varphi }$) converged to a nonzero limit, ∼0.08 (table 1), compared with $\varphi_{0}=0.148$ by equation (10).

The IIM model of figure 6*a* is very similar to the two-species model of figure 1*b* except that here the tree is larger with more species, and serves to highlight the fact that the impact of the misspecification of the model of gene flow is local. The case is also similar to the D-B setting of figure 4, with the only difference that here the hybridizing species *T* had only one descendent species sampled in the data, whereas in figure 4 (D-B) it had two descendent species sampled. Thus estimates of parameters such as the introgression probability and introgression time were similar to those in the D-B setting of figure 4 but with wider CIs (table 1). Unlike approximate methods designed to work with species triplets or quartets only, the Bayesian approach accommodates an arbitrary number of species in the data (with arbitrary data configurations at each locus), so that the difference in taxon sampling has only the effect of affecting the information content in the data.

### Ghost Species

**Fig. 7.**
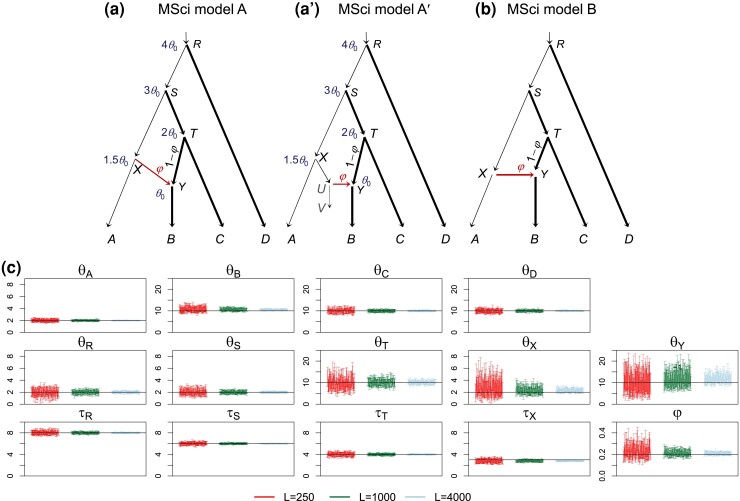
(*a*) MSci model A (fig. 1*A* in Flouri et al. 2020) assumes that $\tau_{X}>\tau_{Y}$ and $\tau_{T}>\tau_{Y}$ and can represent scenario (*a*${}^{\prime }$) in which species *X* split into two species (*A* and *U*), and species *XUV* contributed migrants into species *TYB* at time $\tau_{Y}$ but has since become extinct. This model was used to simulate data, with $\theta_{0}=0.002$ for the thin branches and $\theta_{1}=0.01$ for the thick branches, $\tau_{R}=4\theta_{0},\tau_{S}=3\theta_{0},\tau_{T}=2\theta_{0}$, $\tau_{X}=1.5\theta_{0}$, and $\tau_{Y}=\theta_{0}$. The introgression probability is φ=0.2. The number of sequences is S=4, and the sequence length is n=500. (*b*) MSci model B (fig. 1B in Flouri et al. 2020) used to analyze the data, which incorrectly assumes $\tau_{X}=\tau_{Y}$. (*c*) The 95% HPD CIs for parameters, with θs and τs multiplied by $10^{3}$ and black solid line indicating the true value.

We considered two scenarios in which a species that contributed migrants to extant species has gone extinct or is otherwise unsampled in the data. Note that existence of extinct or unsampled species that *received* genetic materials from ancestors of extant species in the sample is not relevant to the analysis of the sampled data and does not constitute a model misspecification. In the first scenario, model A${}^{\prime }$ of figure 7*a*${}^{\prime }$ is used to simulate data, which assumes that species *XUV* contributed migrants to species *B* but is not included in the sample. Note that this model is equivalent to model A of figure 7*a*. When we fit model B (fig. 7*b*), the only incorrect assumption is the constraint that $\tau_{X}=\tau_{Y}$. This is a minor misspecification. Indeed all parameters shared between the simulation model and the analysis model were well estimated (fig. 7*c*). The estimates of introgression time, ${\hat{\tau }}_{X}={\hat{\tau }}_{Y}\approx 0.0028$ (table 1), were close to the average of the two parameters in the true model (0.0025). Introgression probability $\hat{\varphi }\approx 0.21$ (table 1) was also close to the true value (0.2). The existence of the ghost species (*XUV*) had very little effect on the inference.

**Fig. 8.**
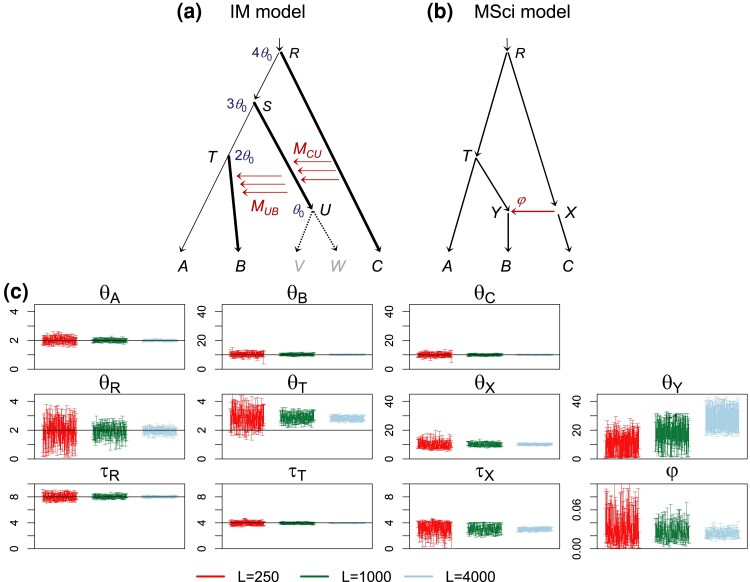
(*a*) Migration model involving ghost species for simulating data. The parameter values used are $\theta_{0}=0.002$ for the thin branches and $\theta_{1}=0.01$ for the thick branches, with the divergence times (τs) shown next to the internal nodes. The number of sequences is S=4, and the sequence length is n=500. The migration rates are $M_{CU}=M_{UB}=0.2$ migrants per generation. (*b*) MSci model used to analyze the data. (*c*) The 95% HPD CI for parameters, with θs and τs multiplied by $10^{3}$, and with black solid lines indicating the true values.

In the second scenario (fig. 8*a*), the true model assumes continuous migration involving intermediate ancestral species that have gone extinct, and the MSci model (fig. 8*b*) was fitted to data sampled from extant species. Divergence times $\tau_{R}$ and $\tau_{T}$ were very well estimated, as were the population sizes shared between the simulation and analysis models ($\theta_{A}$, $\theta_{B}$, $\theta_{C}$, $\theta_{R}$). We expect ${\hat{\tau }}_{T}$ in model B to be dominated by the minimum coalescent time $t_{ab}$ between sequences from *A* and *B*, and this is given by $\tau_{T}^{\left(A\right)}$. Gene flow from branches *RC* to *SU* over the time interval ($\tau_{U},\tau_{S}$) and then from *SU* to *TB* during $\left(\tau_{U},\tau_{T}\right)$ was interpreted as introgression in the MSci model. The effective rate for this migration may be close to $M_{CU}M_{UB}=0.04$, giving $\varphi_{0}=1-e^{-4\times M_{CU}M_{UB}\times (\tau_{T}-\tau_{U})/\theta_{B}}=0.031$. The estimate was ${\hat{\varphi }}_{X}\approx 0.02$ (fig. 8*c*, table 1). The introgression time ${\hat{\tau }}_{X}$ should be between $\tau_{U}=\theta_{0}=0.002$ and $\tau_{T}=2\theta_{0}=0.004$ and the estimate was ≈0.0030 (fig. 8*c*, table 1). Note that both ${\hat{\theta }}_{T}$ and ${\hat{\theta }}_{Y}$ were overestimated (fig. 8*c*). Branch *T* of figure 8*b* corresponds to branches *RS* and *ST* of figure 8*a*, with population size $\theta_{0}=0.002$. Branch *Y* corresponds to a segment of branch *TB* over the time interval $\left(\tau_{U},\tau_{T}\right)$, with $\theta_{1}=0.01$. Overestimation of $\theta_{Y}$ (and $\theta_{T}$) may be because there is a deficit of $t_{bb}$ over the interval $\left(\tau_{U},\tau_{S}\right)$ due to gene flow, and the fitting MSci model, with the amount of gene flow underestimated ($\hat{\varphi }<\varphi_{0}$), used large ${\hat{\theta }}_{Y}$ and ${\hat{\theta }}_{T}$ to compensate.

## Discussion

### The Mode of Gene Flow and the Utility of Misspecified Introgression Models

The asymptotic theory, even though based on only two species with one sequence sampled per species per locus, has been very useful. It generated a number of insights that were confirmed and extended in our simulation. Together the theory and simulation suggest the following correspondence between the MSC-M and MSci models. When gene flow occurs continuously over an extended time period after divergence of two species and we fit the introgression model, the estimated introgression time tends to be closer to the more recent end of the time period of gene flow, because the introgression time is dominated by the most recent coalescent time or the minimum sequence divergence between species. If the true coalescent time is known and used as data, the introgression time will converge to the time when gene flow stopped. At low migration rates ($M<\frac{1}{4}$, say), the species divergence time is correctly estimated by the MSci model, and the introgression probability φ is lower than but close to the expected proportion of migrants ($\varphi^{*}<\varphi_{0}$). The estimate is particularly close under the secondary-contact model (supplementary fig. S4, Supplementary Material online). At very high migration rates, the estimated introgression probability $\varphi^{*}$ may be much less than $\varphi_{0}$, and furthermore the species divergence time is underestimated to account for intermediate coalescent times generated under the MSC-M model. Recent gene flow (as in the SC model) is easier to recover (with $\varphi^{*}$ closer to $\varphi_{0}$) than ancient gene flow (as in the IIM model).

The accurate estimation of species divergence times under the MSci model despite the misspecification, at least at lower migration rates (e.g., $\tau_{R}$ for M≤0.3 in fig. 3), may be worth emphasizing. It is well known that ignoring gene flow between two species may lead to serious underestimation of the species divergence time. Here our results suggest that if gene flow is continuous, the MSci model assuming introgression at a fixed time point still gives reliable estimates of the species divergence time. The estimated introgression probability (φ) may also serve as a useful guide even though it reflects both the migration rate per generation (*m* or *M*) and the time duration of the period of gene flow (eq. 10). Even if gene flow occurs continuously over time (so that the migration model is a more realistic model), the MSci model is effective in extracting historical information about species divergence times and population sizes. Note that on the evolutionary time scale, a few hundred or thousand generations may count as a fixed time point, in which case the MSci model may provide an adequate approximation.

Both the asymptotic theory and simulation have highlighted the semi-unidentifiability or confounding effects between the introgression probability (φ) and the population size of the donor species ($\theta_{S}$ in fig. 1*d*) (e.g., fig. 2, methods c and d). The problem is particularly acute under the IIM model applied to small datasets (with short loci, few sequences per species, or few loci), where high estimates of φ with wide CIs are produced even though migration occurs at very low rates (fig. 3). One such case has been observed in a recent analysis of genomic data from the *erato* group of *Heliconius* butterflies (Thawornwattana et al. 2022). The estimated *H. sara*→*H. demeter* introgression probability was high with wide CIs for some chromosomal regions with a small number of loci (e.g., chromosome 21 with 4350 noncoding and 3628 coding loci, and an inversion on chromosome 15 with 149 noncoding and 167 coding loci), with the introgression time close to the species divergence time, whereas for the other large chromosomes, the estimates were nearly zero ($\hat{\varphi }<0.01$). The true rate in this case appeared to be φ≈0, but the limited data from small chromosomal segments led to poorly supported large introgression rates, as in our simulations (fig. 3).

We demonstrated that including multiple samples from the same species (in particular, from recipient species) is important to resolving unidentifiability issues or confounding effects, as well as boosting up the information content concerning the rate of gene flow in the data. In this regard, it may be noted that many approximate methods are designed to use only one sample per species, and it has been claimed that “adding more samples provides little new information with respect to introgression” (Hibbins and Hahn 2022). We suggest that this may not be a generally correct statement.

Overall, our simulations using larger species trees with more than two species suggest that misspecification of the mode of gene flow (continuous migration versus episodic hybridization/introgression) has relatively small and localized effects, restricted to divergence times and population sizes around the lineages involved in gene flow, while species divergence times, population sizes for extant species and for ancestral species not involved in gene flow are largely unaffected. If gene flow occurs between species *A* and *B* but more distantly related species are included in the data sample, parameters outside the *AB* clade are largely unaffected (e.g., compare results for the IIM model for two species of fig. 3 with those for three species of fig. 6). Similarly, if *A* represents a clade rather than one species, divergence times and population sizes inside the *A* clade are not affected by gene flow involving the branch ancestral to the *A* clade (e.g., compare the D-B setting of fig. 4 with the IIM model of fig. 3).

Assigning gene flow to parental or daughter branches causes the introgression probability to be underestimated, and the introgression time to collapse onto the species divergence time. This result may be used to diagnose the mis-assignment of introgression lineages in real data analysis (Ji et al. 2022). A number of authors have discussed the impact of ghost species on detection of between-species gene flow (Beerli 2004; Ottenburghs 2020). Tricou et al. (2022) used simulations to demonstrate that *D*-statistics can be misled to detect false signals of introgression when the model involved an unsampled (ghost) species. In our simulations, the impact of ghost species on Bayesian estimation of introgression rate and time was minor provided we considered the rate of gene flow in the migration and introgression models to reflect both indirect gene flow via intermediate species and direct gene flow.

### Testing Models of Gene Flow

In this study, we fixed the model of introgression in our analyses, with all introgression events pre-identified, to examine the effects of model misspecification. One may ask what happens if different introgression models (which for example assign introgression events onto different branches of the species tree) are compared using genomic data. Currently, both *beast and Phylonet have implemented cross-model MCMC algorithms under the MSci model, which insert and delete introgression events on the species tree, allowing the Markov chain to move between models. Those algorithms are computationally expensive and currently the two programs can handle only very small datasets (with <100 loci, say). In the Bpp program, one may use the Bayes factor to compare two MSci models, using thermodynamic integration (Gelman and Meng 1998; Lartillot and Philippe 2006) combined with Gaussian quadrature to calculate the marginal likelihood values (Rannala and Yang 2017). In the case where the compared models are nested (e.g., one with introgression and another without), the Bayes factor may also be calculated through the Savage–Dickey density ratio (Dickey 1971), which uses only a within-model MCMC run under the more general model (Ji et al. 2022). This has a computational advantage over reversible jump MCMC (Green 1995), and has recently been applied to formulate and compare introgression models in an analysis of genomic data from the *Tamias quadrivittatus* group of North American chipmunks (Ji et al. 2022). Calculation of marginal likelihood values or Bayes factors may be feasible if we have only a small number of well-specified models but may not be feasible for searching in the space of MSci models for a given set of species.

Approximate methods have also been developed to infer introgression events or the so-called phylogenetic networks using summaries of the multilocus sequence data. For example, estimated gene tree topologies may be treated as data, as in Phylonet/gt (Wen et al. 2016). Some methods are designed to detect gene flow in a small tree with three or four species, including summary methods based on genome-wide site-pattern counts (such as *D* and Hyde discussed earlier) or on estimated gene trees (e.g., Snaq) and maximum likelihood applied to multilocus sequence alignments (e.g., 3s, Zhu and Yang 2012; Dalquen et al. 2017). Results for species subsets may then be combined to formulate an introgression model on the large tree for all species, which is a challenging task (Edelman et al. 2019; Thawornwattana et al. 2022). In summary, there is currently an acute need for improving the computational efficiency of Bayesian MCMC algorithms for inference under the MSC model with gene flow and the statistical efficiency of approximate methods.

It will also be interesting to use the same genomic data to compare the MSC-M and MSci models. The two classes of models often predict very different distributions of gene trees and coalescent times (e.g., supplementary figs. S1, S3, S5, Supplementary Material online; see also Jiao and Yang 2021). Thus, genomic data may be informative to distinguish them. A stochastic search in the combined space of MSC-M and MSci models may be infeasible, as the two types of models are very different. However, they can be compared using Bayes factors.

## Materials and Methods

### Simulation to Establish a Correspondence between the Migration and Introgression Models in the Case of Two Species

We analyzed the relationships between parameters when data are generated under the continuous migration model (IM, IIM, and SC; fig. 1*a*–*c*) and analyzed under the episodic introgression (MSci) model (fig. 1*d*). Our theory assumed an infinite number of loci (L=∞), a finite number of sites per sequence (*n*), with only one sequence per species per locus. We conducted computer simulations to augment the theoretical analysis. Data of multilocus sequence alignments were simulated under the IM, IIM, and SC models of figure 1*a*–*c*, and analyzed under the MSci model (fig. 1*d*). Population sizes on the species tree (fig. 1) were $\theta_{0}=0.002$ for the thin branches and $\theta_{1}=0.01$ for the thick branches. Migration occurred from species *A* to *B* after their divergence at $\tau_{R}=\theta_{0}$ in the IM model, between $\tau_{R}=2\theta_{0}$ and $\tau_{T}=\theta_{0}$ in the IIM model, and between $\tau_{T}=\theta_{0}$ and the present time in the SC model. In the standard model, the migration rate was $M_{AB}=0.2$ individuals per generation. Each dataset consisted of L=4,000 loci, with S=4 sequences per species, and n=1,000 sites per sequence. We conducted four sets of simulation to examine the impact of the number of sites per sequence (*n*), the number of sequences per species (*S*), the number of loci (*L*), and the migration rate ($M_{AB}$). The values used were n=250, 1,000, 4,000, 16,000, 64,000; S=1, 2, 4, 8, 16; L=250, 500, 1,000, 2,000, 4,000, 8,000; and $M_{AB}=0.01$, 0.02, 0.03, 0.04, 0.05, 0.07, 0.1, 0.2, 0.3, 0.4, 0.5, 0.7, 1.0, 1.5, 2.0. With three models (IM, IIM, and SC), four factors (n,S,L,M), and 30 replicates, a total of 3×(5+5+6+13)×30=2,790 datasets were simulated. Data were simulated using Bpp 4.4.1 (Flouri et al. 2018, 2020), by generating the gene tree with coalescent times for each locus and then “evolving” sequences along branches of the gene tree under the JC mutation model (Jukes and Cantor 1969). Sequences at the tips of the gene tree constituted the data at the locus.

Each dataset was analyzed using Bpp under the MSci model (fig. 1*d*) to estimate the parameters. This is the so-called A00 analysis, with the model fixed (Yang 2015). The Bayesian implementation of the MSci model in Bpp accommodates gene-tree reconstruction uncertainties while making use of information in both gene tree topologies and branch lengths, and allows the estimation of the direction, timing, and strength of introgression (Jiao et al. 2021). The JC mutation model was assumed in the analysis. Gamma priors were assigned to population size parameters (θ) and to the age of the root on the species tree; θ∼G(2,400) and $\tau_{0}∼G(2,200)$. Note that the gamma distribution G(a,b) has mean a/b and variance $a/b^{2}$, so that the shape parameter a=2 means diffuse priors. Introgression probability φ was assigned the beta prior beta(1,1), which is U(0,1).

We used 32,000 MCMC iterations as burnin, and took $2\times 10^{5}$ samples, sampling every five iterations.

### Introgression Events Assigned to Wrong Branches

Data were simulated under models A and B of figure 4 and analyzed under models A and B, possibly with the introgression event assigned incorrectly onto either the parental or a daughter branch of the branch truly involved in introgression. The species divergence times (τ) are shown in the trees (fig. 4). We used S=4 sequences per species per locus, with n=500 sites in the sequence. The number of loci was L=250, 1,000, and 4,000. We used two population sizes, with $\theta_{0}=0.002$ for the thin branches and $\theta_{1}=0.01$ for the thick branches. The number of replicates was 100.

Each dataset was analyzed using Bpp under both models A and B (fig. 4*a*and*b*). Gamma priors were assigned to parameters, θ∼G(2,400) with mean 0.005 and $\tau_{0}∼G(2,200)$ with mean 0.01. With two trees/models, three numbers of loci, 2×3×100=600 datasets were simulated, each analyzed under models A and B. We used 32,000 MCMC iterations as burnin, and took $2\times 10^{5}$ samples, sampling every five iterations.

### Continuous Migration versus Episodic Introgression

Data were simulated under the MSC-M models C and D of figure 4*c*and*d*, with continuous migration at the rate M=0.1 migrants per generation, and analyzed under MSci models A and B (fig. 4*a*and*b*), resulting in four settings: C-A (simulation model C and analysis model A), C-B, D-A, and D-B. In setting C-A and D-B, gene flow was continuous in the true model but the MSci model assumes episodic introgression at a particular time point, so that the mode of gene flow is misspecified. In settings C-B and D-A, the mode of gene flow was similarly misspecified but we had in addition mis-assignment of gene flow to wrong branches on the species tree. Other parameter settings were the same as above. With two trees, three numbers of loci (*L*), a total of 600 datasets were generated, each analyzed twice (under models A and B).

### Isolation with Initial Migration (IIM) Model

Data were simulated under the IIM model A of figure 6*a*, with A→B migration over the time period $\left(\tau_{T},\tau_{S}\right)$, and analyzed under the MSci model of figure 6*b*, assuming introgression at time $\tau_{X}=\tau_{Y}$. The IIM model was specified using a ghost species (*U*) from which no sequences were available. We generated 100 replicate datasets, each of L=250, 1,000, or 4,000 loci, with a total of 300 datasets simulated. MCMC settings were the same as above.

### Ghost Species

To assess the effects of unsampled ghost population, we simulated data under MSci model A${}^{\prime }$ (see fig. 1*A* in Flouri et al. 2020) of figure 7*a*${}^{\prime }$ and analyzed them under the MSci model B of figure 7*b*, with $\tau_{X}=\tau_{Y}$ incorrectly assumed. Here introgression involved a ghost species *XUV* which went extinct or was otherwise unsampled in the data. This scenario is equivalent to model A of figure 7*a*. With the three values for *L* (250, 1,000, 4,000), 300 datasets were generated, all analyzed under the MSci model (fig. 7*b*).

We also used the IIM model of figure 8*a* to generate data, with migration from species *RC* to *SU* and from *SU* to *TB*, and with *V* and *W* to be unsampled ghost species. Data (i.e., sequences from A,B and *C*) were analyzed under the MSci model of figure 8*b*. We used three values for *L* (250, 1,000, 4,000) and 100 replicates, with 300 datasets simulated in total. Other settings were the same as above.

## Supplementary Material

msac237_Supplementary_Data
