# Supplementary material for: Inference of Gene Flow between Species under Misspecified Models
Source: Mol Biol Evol. 2022 Nov 1;39(12):msac237. doi: 10.1093/molbev/msac237 (PMC9729068; doi:10.1093/molbev/msac237)
Supplement: msac237_Supplementary_Data [file msac237_supplementary_data.pdf]

Online Supplemental Information for
"Inference of gene flow between species under misspecified models"
Huang et al.

SI text. Likelihood function under the IM and MSci models in the case of two species

Here we derive the likelihood function under the three continuous migration models (IM, IIM, SC) and the episodic introgression (MSci) model for two species (fig. 1**a-d**) when the data consist of an infinite number of loci, with two sequences sampled at each locus, one from each species. The data at each locus can be summarized as x differences at n sites. The infinite-sites mutation model is assumed so that the probability of data given the coalescent time is given by the Poisson probability (eq. 8). To calculate the likelihood, we integrate over the unknown coalescent time t, which has density $f_{\rm m}(t|\Theta_{\rm m})$ (eq. 3) under the IM or IIM model, $f_{\rm sc}(t|\Theta_{\rm m})$ (eq. 4) under the SC model, and $f_{\rm i}(t|\Theta_{\rm i})$ (eq. 5) under the MSci model (Wilkinson-Herbots, 2008, 2012; Costa and Wilkinson-Herbots, 2021).

Under the IM and IIM models (fig. 1**a&b**), we have from eq. 3

$$f(x|\Theta_{\rm m}) = \int_0^\infty f(x|t) f_{\rm m}(t|\Theta_{\rm m})\,{\rm d}t = \int_0^\infty \frac{1}{x!}(2nt)^x\,{\rm e}^{-2nt} f_{\rm m}(t|\Theta_{\rm m})\,{\rm d}t = I_1 + I_2. \tag{S1}$$

The first term is

$$\begin{aligned} I_1 &= \int_{\tau_T}^{\tau_R} \frac{1}{x!}(2nt)^x\,{\rm e}^{-2nt}\,\frac{2w}{2-w\theta_A}\left[{\rm e}^{-w(t-\tau_T)} - {\rm e}^{-\frac{2}{\theta_A}(t-\tau_T)}\right]{\rm d}t \\ &= \frac{(2n)^x}{x!}\,\frac{2w}{2-w\theta_A}\left[{\rm e}^{w\tau_T}\int_{\tau_T}^{\tau_R} t^x\,{\rm e}^{-(2n+w)t}\,{\rm d}t - {\rm e}^{\frac{2}{\theta_A}\tau_T}\int_{\tau_T}^{\tau_R} t^x\,{\rm e}^{-(2n+\frac{2}{\theta_A})t}\,{\rm d}t\right], \end{aligned} \tag{S2}$$

where the two integrals are

$$\begin{aligned} \int_{\tau_T}^{\tau_R} t^x\,{\rm e}^{-(2n+w)t}\,{\rm d}t &= \frac{1}{(2n+w)^{x+1}}\Big[\gamma(x+1,\tau_R(2n+w)) - \gamma(x+1,\tau_T(2n+w))\Big], \\ \int_{\tau_T}^{\tau_R} t^x\,{\rm e}^{-(2n+\frac{2}{\theta_A})t}\,{\rm d}t &= \frac{1}{(2n+\frac{2}{\theta_A})^{x+1}}\Big[\gamma(x+1,\tau_R(2n+\tfrac{2}{\theta_A})) - \gamma(x+1,\tau_T(2n+\tfrac{2}{\theta_A}))\Big], \end{aligned} \tag{S3}$$

with

$$\Gamma(a) = \int_0^\infty t^{a-1}\,{\rm e}^{-t}\,{\rm d}t, \quad \gamma(a,x) = \int_0^x t^{a-1}\,{\rm e}^{-t}\,{\rm d}t, \tag{S4}$$

to be the gamma function and the lower incomplete gamma function, respectively, with $\gamma(a,\infty) = \Gamma(a)$.

Similarly the second term in eq. S1 is

$$\begin{aligned} I_2 &= \int_{\tau_R}^\infty \frac{1}{x!}(2nt)^x\,{\rm e}^{-2nt}\,\frac{1}{2-w\theta_A}\left[2\,{\rm e}^{-w(\tau_R-\tau_T)} - w\theta_A\,{\rm e}^{-\frac{2}{\theta_A}(\tau_R-\tau_T)}\right]\frac{2}{\theta_R}\,{\rm e}^{-\frac{2}{\theta_R}(t-\tau_R)}\,{\rm d}t \\ &= \frac{(2n)^x}{x!}\,\frac{1}{2-w\theta_A}\left[2\,{\rm e}^{-w(\tau_R-\tau_T)} - w\theta_T\,{\rm e}^{-\frac{2}{\theta_A}(\tau_R-\tau_T)}\right]\frac{2}{\theta_R}\,{\rm e}^{\frac{2}{\theta_R}\tau_R}\int_{\tau_R}^\infty t^x\,{\rm e}^{-(2n+\frac{2}{\theta_R})t}\,{\rm d}t \\ &= \frac{(2n)^x}{x!}\,\frac{1}{2-w\theta_A}\left[2\,{\rm e}^{-w(\tau_R-\tau_T)} - w\theta_T\,{\rm e}^{-\frac{2}{\theta_A}(\tau_R-\tau_T)}\right]\frac{2}{\theta_R}\,{\rm e}^{\frac{2}{\theta_R}\tau_R} \times \frac{\Gamma(x+1) - \gamma(x+1,\tau_R(2n+\frac{2}{\theta_R}))}{(2n+\frac{2}{\theta_R})^{x+1}}. \end{aligned} \tag{S5}$$

Putting everything together, we get

$$\begin{aligned} f_{\rm m}(x|\Theta_{\rm m}) = {} & \frac{(2n)^x}{x!}\,\frac{2w}{2-w\theta_A}\Bigg(\frac{{\rm e}^{w\tau_T}}{(2n+w)^{x+1}}\big[\gamma(x+1,\tau_R(2n+w)) - \gamma(x+1,\tau_T(2n+w))\big] \\ & - \frac{{\rm e}^{\frac{2}{\theta_A}\tau_T}}{(2n+\frac{2}{\theta_A})^{x+1}}\Big[\gamma(x+1,\tau_R(2n+\tfrac{2}{\theta_A})) - \gamma(x+1,\tau_T(2n+\tfrac{2}{\theta_A}))\Big]\Bigg) \\ & + \frac{(2n)^x}{x!}\,\frac{1}{2-w\theta_A}\left[2\,{\rm e}^{-w(\tau_R-\tau_T)} - w\theta_A e^{-\frac{2}{\theta_A}(\tau_R-\tau_T)}\right]\frac{2}{\theta_R}\,{\rm e}^{\frac{2}{\theta_R}\tau_R} \\ & \times \frac{\Gamma(x+1) - \gamma(x+1,\tau_R(2n+\frac{2}{\theta_R}))}{(2n+\frac{2}{\theta_R})^{x+1}}. \end{aligned} \tag{S6}$$

Similarly, under the secondary-contact (SC) model (fig. 1**c**), the density of coalescent time t is given in eq. 4. The

probability of observing x differences at n sites at a locus is

$$f_{\text{sc}}(x|\Theta_{\text{m}}) = \int_0^\infty f(x|t) f_{\text{sc}}(t|\Theta_{\text{m}})\,\mathrm{d}t = J_1 + J_2 + J_3, \tag{S7}$$

where

$$\begin{aligned}
J_1 &= \int_0^{\tau_T} \frac{1}{x!}(2nt)^x \mathrm{e}^{-2nt} \frac{w\theta_A}{2-w\theta_A}\left[\mathrm{e}^{-wt} - \mathrm{e}^{-\frac{2}{\theta_A}t}\right] \tfrac{2}{\theta_A}\,\mathrm{d}t \\
&= \frac{(2n)^x}{x!} \frac{2w}{2-w\theta_A}\left[\frac{\gamma(x+1, \tau_T(2n+w))}{(2n+w)^{x+1}} - \frac{\gamma(x+1, \tau_T(2n+\frac{2}{\theta_A}))}{(2n+\frac{2}{\theta_A})^{x+1}}\right], \\
J_2 &= \int_{\tau_T}^{\tau_R} \frac{1}{x!}(2nt)^x \mathrm{e}^{-2nt} \frac{w\theta_A}{2-w\theta_A}\left[\mathrm{e}^{-w\tau_T} - \mathrm{e}^{-\frac{2}{\theta_A}\tau_T}\right] \tfrac{2}{\theta_A}\mathrm{e}^{-\frac{2}{\theta_A}(t-\tau_T)}\,\mathrm{d}t \\
&= \frac{(2n)^x}{x!} \frac{2w}{2-w\theta_A}\left[\mathrm{e}^{-w\tau_T} - \mathrm{e}^{-\frac{2}{\theta_A}\tau_T}\right] \frac{\mathrm{e}^{\frac{2}{\theta_A}\tau_T}}{(2n+\frac{2}{\theta_A})^{x+1}}\left[\gamma(x+1, \tau_R(2n+\tfrac{2}{\theta_A})) - \gamma(x+1, \tau_T(2n+\tfrac{2}{\theta_A}))\right], \\
J_3 &= \int_{\tau_R}^{\infty} \frac{1}{x!}(2nt)^x \mathrm{e}^{-2nt}\left[\frac{w\theta_A}{2-w\theta_A}\left(\mathrm{e}^{-w\tau_T} - \mathrm{e}^{-\frac{2}{\theta_A}\tau_T}\right)\mathrm{e}^{-\frac{2}{\theta_A}(\tau_R-\tau_T)} + \mathrm{e}^{-w\tau_T}\right] \tfrac{2}{\theta_R}\mathrm{e}^{-\frac{2}{\theta_R}(t-\tau_R)}\,\mathrm{d}t \\
&= \frac{(2n)^x}{x!}\left[\frac{w\theta_A}{2-w\theta_A}\left(\mathrm{e}^{-w\tau_T} - \mathrm{e}^{-\frac{2}{\theta_A}\tau_T}\right)\mathrm{e}^{-\frac{2}{\theta_A}(\tau_R-\tau_T)} + \mathrm{e}^{-w\tau_T}\right] \tfrac{2}{\theta_R} \frac{\mathrm{e}^{\frac{2}{\theta_R}\tau_R}}{(2n+\frac{2}{\theta_R})^{x+1}}\left[\Gamma(x+1) - \gamma(x+1, \tau_R(2n+\tfrac{2}{\theta_R}))\right].
\end{aligned} \tag{S8}$$

Finally, under the MSci model (fig. 1**d**), the density of coalescent time t is given in eq. 5. We have

$$\begin{aligned}
f_{\text{i}}(x|\Theta_{\text{i}}) &= \int_0^\infty f(x|t) f_{\text{i}}(t|\Theta_{\text{i}})\,\mathrm{d}t \\
&= \int_{\tau_S}^{\tau_R} \frac{1}{x!}(2nt)^x \mathrm{e}^{-2nt}\,\varphi \tfrac{2}{\theta_S}\mathrm{e}^{-\frac{2}{\theta_S}(t-\tau_S)}\,\mathrm{d}t + \int_{\tau_R}^{\infty} \frac{1}{x!}(2nt)^x \mathrm{e}^{-2nt}\left[\varphi\,\mathrm{e}^{-\frac{2}{\theta_S}(\tau_R-\tau_S)} + (1-\varphi)\right] \tfrac{2}{\theta_R}\mathrm{e}^{-\frac{2}{\theta_R}(t-\tau_R)}\,\mathrm{d}t \\
&= \frac{(2n)^x}{x!}\varphi\frac{2}{\theta_S}\mathrm{e}^{\frac{2}{\theta_S}\tau_S}\int_{\tau_S}^{\tau_R} t^x \mathrm{e}^{-(2n+\frac{2}{\theta_S})t}\,\mathrm{d}t + \frac{(2n)^x}{x!}\left[\varphi\,\mathrm{e}^{-\frac{2}{\theta_S}(\tau_R-\tau_S)} + (1-\varphi)\right]\tfrac{2}{\theta_R}\mathrm{e}^{\frac{2}{\theta_R}\tau_R}\int_{\tau_R}^{\infty} t^x \mathrm{e}^{-(2n+\frac{2}{\theta_R})t}\,\mathrm{d}t \\
&= \frac{(2n)^x}{x!}\varphi\frac{2}{\theta_S}\mathrm{e}^{\frac{2}{\theta_S}\tau_S} \times \frac{\gamma(x+1, \tau_R(2n+\frac{2}{\theta_S})) - \gamma(x+1, \tau_S(2n+\frac{2}{\theta_S}))}{(2n+\frac{2}{\theta_S})^{x+1}} \\
&\quad + \frac{(2n)^x}{x!}\left[\varphi\,\mathrm{e}^{-\frac{2}{\theta_S}(\tau_R-\tau_S)} + (1-\varphi)\right]\tfrac{2}{\theta_R}\mathrm{e}^{\frac{2}{\theta_R}\tau_R} \times \frac{\Gamma(x+1) - \gamma(x+1, \tau_R(2n+\frac{2}{\theta_R}))}{(2n+\frac{2}{\theta_R})^{x+1}}.
\end{aligned} \tag{S9}$$

References

Costa, R. J. and Wilkinson-Herbots, H. M. 2021. Inference of gene flow in the process of speciation: Efficient maximum-likelihood implementation of a generalised isolation-with-migration model. *Theor. Popul. Biol.*, 140(1–15).

Wilkinson-Herbots, H. M. 2008. The distribution of the coalescence time and the number of pairwise nucleotide differences in the "isolation with migration" model. *Theor. Popul. Biol.*, 73: 277–288.

Wilkinson-Herbots, H. M. 2012. The distribution of the coalescence time and the number of pairwise nucleotide differences in a model of population divergence or speciation with an initial period of gene flow. *Theor. Popul. Biol.*, 82: 92–108.

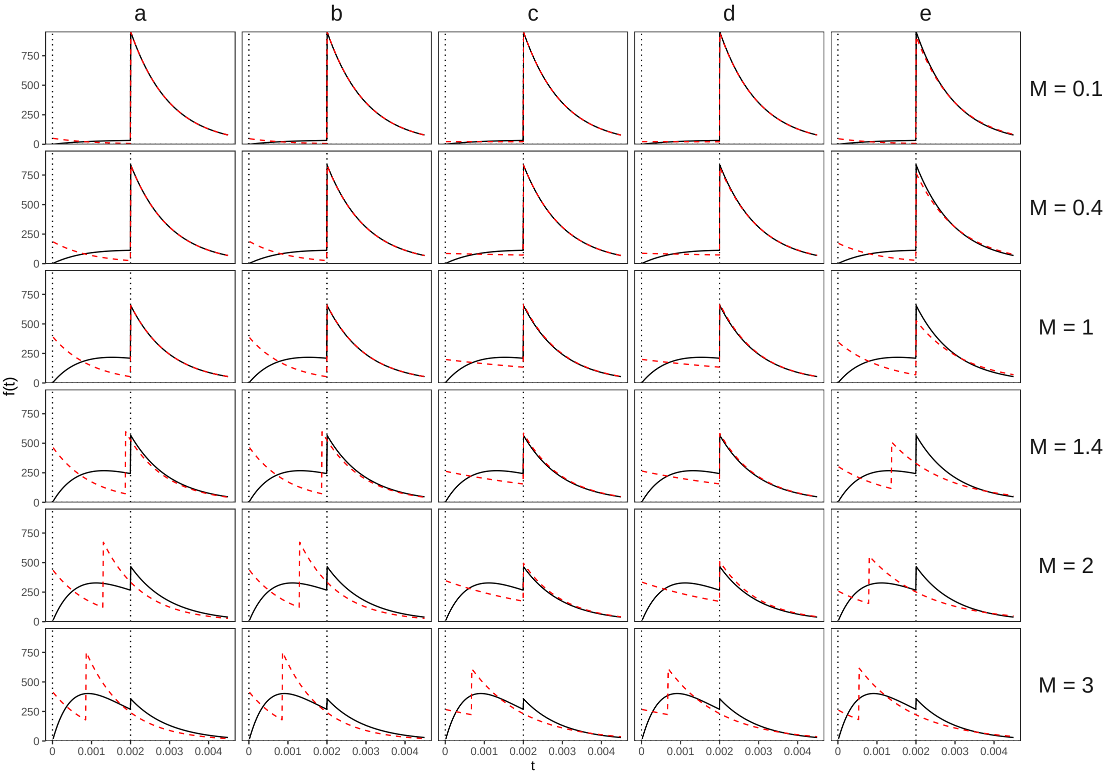

Figure S1: **(kl-IM:ft)** The true distribution of coalescent time $f_{\mathrm{m}}(t)$ under the IM model (black; fig. 1**a**) and the best-fitting distribution $f_{\mathrm{i}}(t)$ under the MSci model (red; fig. 1**d**). See figure 2 for the methods of analysis (a-e) and for the MLEs. Note that the discontinuity points in the fitting distribution reflect the MLEs of divergence times ($\tau_S^* = 0$ and τ_R^*). The true distribution depends on M but is the same for different methods of analysis (a-e).

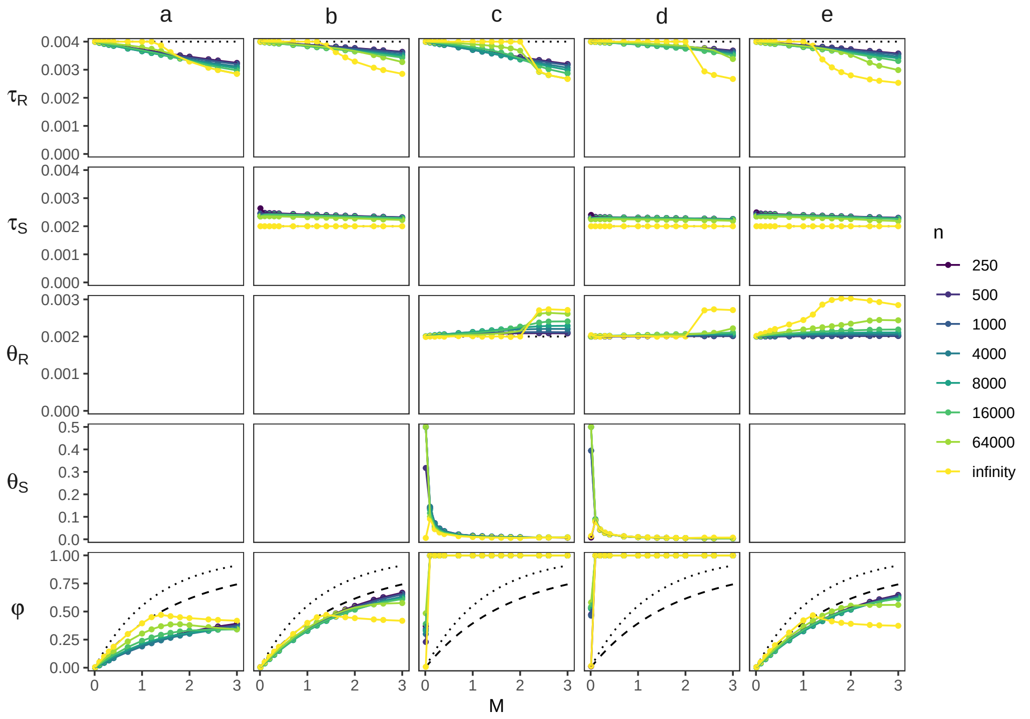

Figure S2: **(kl-IIM:MLE)** Best-fitting parameter values under the MSci model of figure 1**d** when data of two sequences per locus (each of n sites) are generated under the IIM model of figure 1**a**. See legend to figure 2 for the description of the five methods (a-e). In (a) and (c), τ_S is fixed at τ_T, while in (e), the constraint $\theta_R = \theta_S$ is imposed. The true and best-fitting distributions of the coalescent time (t) are in figure S3.

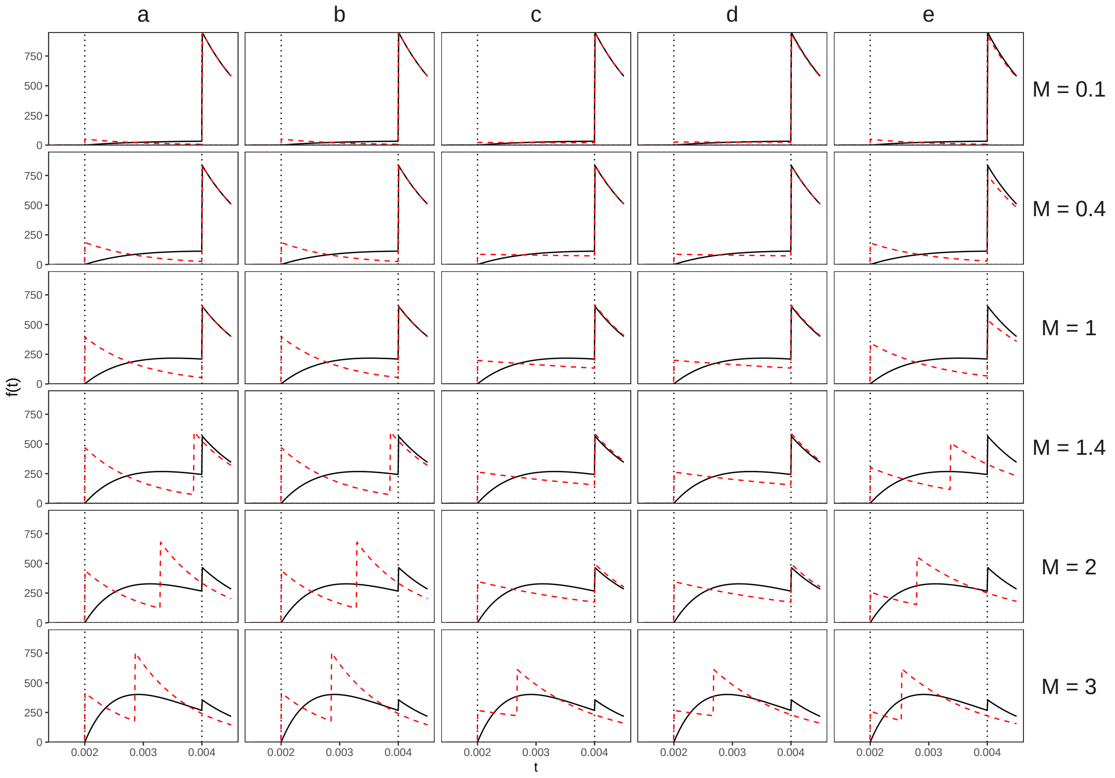

Figure S3: **(kl-IIM:ft)** The true distribution of coalescent time $f_{\mathrm{m}}(t)$ under the IIM model (black; fig. 1**b** with $\tau_T > 0$) and the best-fitting distribution $f_{\mathrm{i}}(t)$ under the MSci model (red; fig. 1**d**). The MLEs are shown in figure S2.

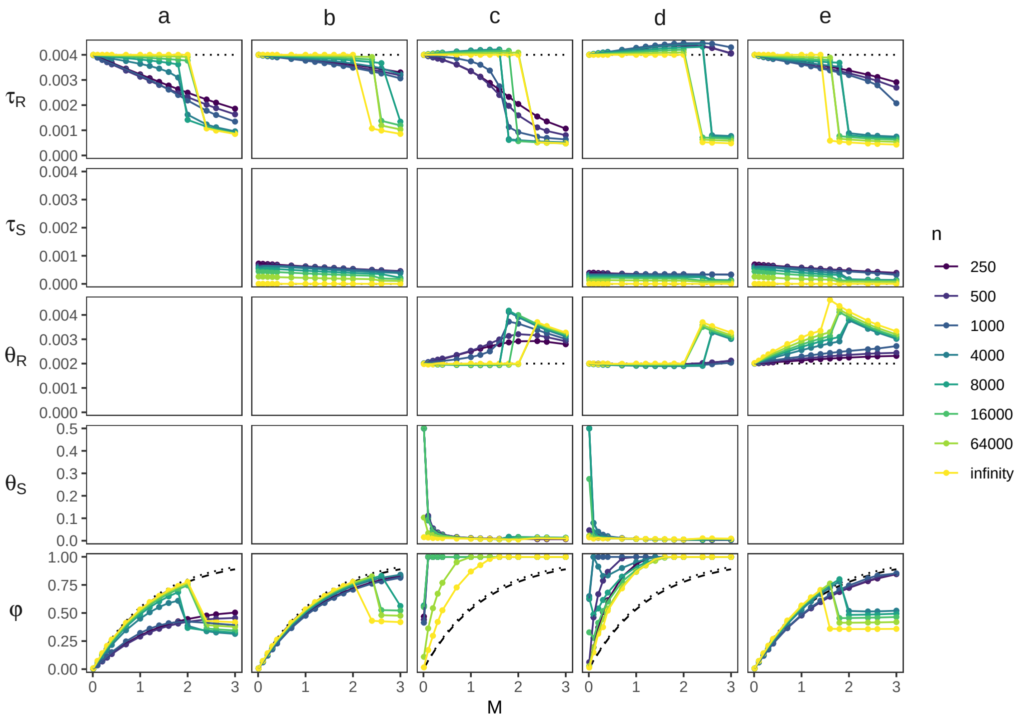

Figure S4: **(kl-SC:MLE,** $\tau_R = 2\theta_0, \tau_T = \theta_0$) Best-fitting parameter values under the MSci model of figure 1**d** when data of two sequences per locus (each of n sites) are generated under the SC model of figure 1**c**. See figure 1 for parameter values used. See legend to figure 2 for the description of the five methods (a-e). In (a) and (c), $\tau_S = 0$ is fixed, while in (e), the constraint $\theta_R = \theta_S$ is imposed. The true and best-fitting distributions of the coalescent time (t) are in figure S5.

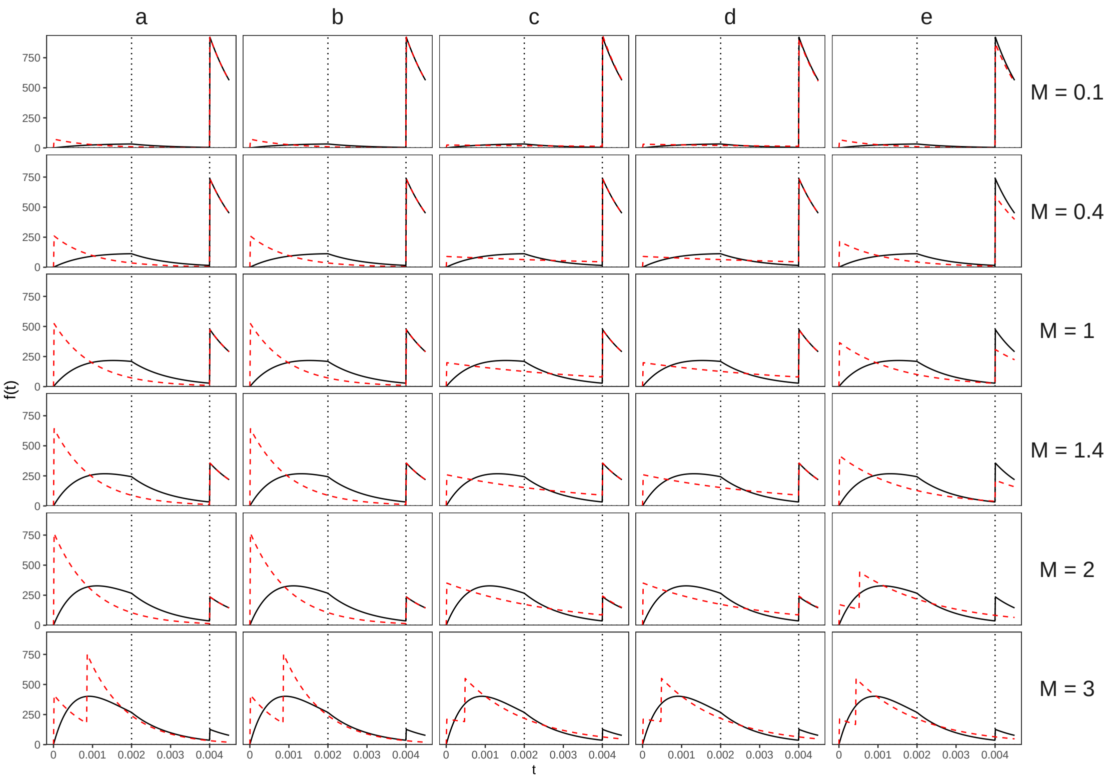

Figure S5: **(kl-SC:ft)** The true distribution of coalescent time $f_\mathrm{m}(t)$ under the SC model (black; fig. 1**c**) and the best-fitting distribution $f_\mathrm{i}(t)$ under the MSci model (red; fig. 1**d**). The MLEs are shown in figure S4.

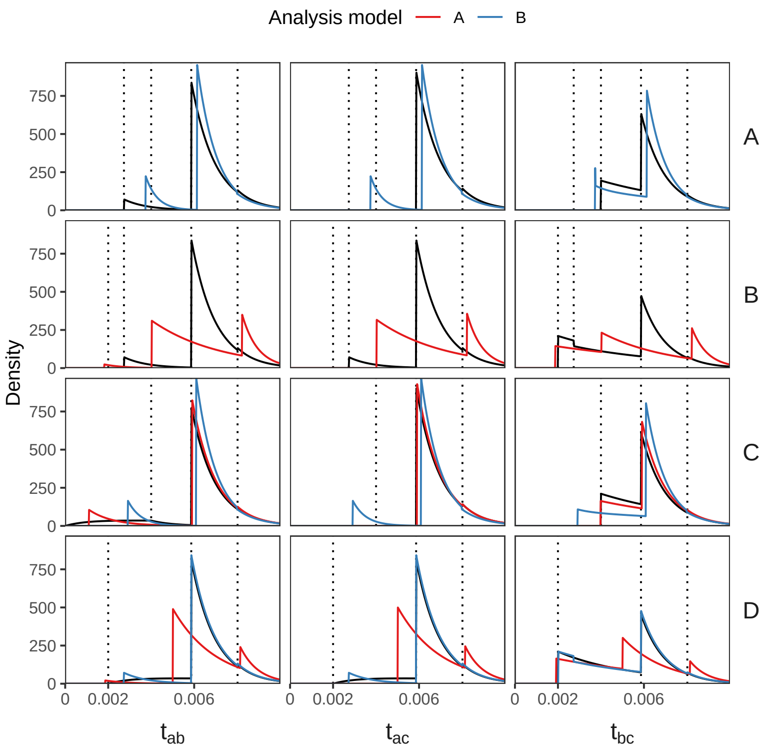

Figure S6: The true (black) and fitting (red and blue) distributions of coalescent times between sequences from two species, $f(t_{ab}), f(t_{ac}), f(t_{bc})$, when data are generated under models A, B, C, and D of figure 4 and analyzed under models A and B. The row corresponds to the true model (A, B, C, or D) while the colour lines indicate the fitting models (A and B). For example, the first row corresponds to the A-B setting, and the second row the B-A setting (fig. 4), with the discontinuity points in the fitting models corresponding to τ_T, τ_X and τ_S. The fitting distributions are calculated using parameter estimates (averages of posterior means) from the simulated data with $L = 4000$ loci (fig. 4**e**).

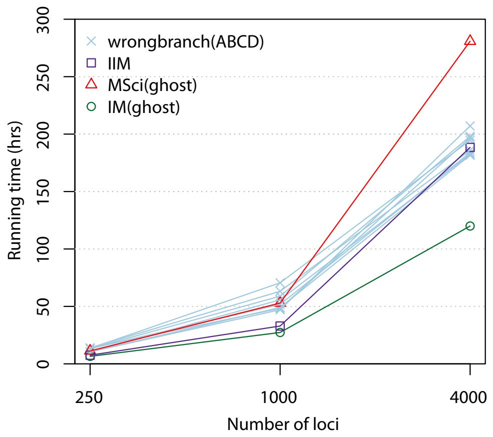

Figure S7: Average BPP running time over replicate datasets for different parameter settings and different numbers of loci. Each run uses one thread.
